# Nanometric mineral inclusions from a fluid-rich diamond: identification, structure, and implications for deep Earth

**DOI:** 10.1038/s41467-026-74619-3

**Published:** 2026-06-23

**Authors:** Yanjuan Wang, Fabrizio Nestola, Fernando Cámara, Maxwell C. Day, Francesca Innocenzi, Matteo Ardit, Robert W. Luth, D. Graham Pearson, Yaakov Weiss, Suzette Timmerman, Martha G. Pamato, Davide Novella, Nicola Rotiroti, Jürgen Grässlin, Christian J. Schürmann, Anna Barbaro, Kai Qu, Claudia Agnini, Zengqian Hou

**Affiliations:** 1 State Key Laboratory of Deep Earth and Mineral Exploration, Chinese Academy of Geological Sciences, Beijing, China; 2 Dipartimento di Geoscienze, Università degli Studi di Padova, Via Gradenigo 6, Padova, Italy; 3 Dipartimento di Scienze della Terra, Università degli Studi di Milano, Via Luigi Mangiagalli 34, Milan, Italy; 4 Department of Earth and Atmospheric Sciences, University of Alberta, 116 St & 85 Ave, Edmonton, AB Canada; 5 Institute of Earth Sciences, The Hebrew University of Jerusalem, Jerusalem, Israel; 6 Institute of Geological Sciences, University of Bern, Baltzerstrasse 1 + 3, Bern, Switzerland; 7 Rigaku Europe SE, Hugenottenallee 167, Neu-Isenburg, Germany; 8 Tianjin Centre, China Geological Survey, Tianjin, China; 9 School of Earth Sciences and Engineering, Nanjing University, Nanjing, China

**Keywords:** Mineralogy, Geochemistry

## Abstract

Fluid-rich (cloudy/fibrous) diamonds host millions of micrometric fluid inclusions that can reveal the nature of diamond-forming media. Mineral inclusions in fluid-rich diamonds are nano to micrometric making structural and chemical characterization of the different phases difficult. Consequently, limited work has been done determining the pressures, temperatures and depths at which such inclusions form. The relationship between such conditions and those of fluid-rich diamond formation remains unclear. We report the use of micro-electron diffractometry to identify and anisotropically refine the structure of a nanometric åkermanite inclusion in a fluid-rich diamond from South Africa. Additional nanometric Ba/Sr-carbonate inclusions were detected. FTIR analyses revealed a highly aggregated, gem-quality core surrounded by a rim rich in high-density fluid (HDF) inclusions from which åkermanite crystallized. Åkermanite formed during HDF depressurization due to kimberlite eruption or exhumation. In the latter scenario, åkermanite constrained diamond formation to a minimum temperature of 1000 °C at ∼140 km depth (~4.6 GPa). Analysis of the HDFs reveal low-Mg carbonatitic to silicic compositions. The high BaO and Cl and the δ^13^C values of the diamond (−5.72 to −7.84 ‰) are attributed to formation via penetration of saline fluid into an eclogite, related to subduction of carbonated altered oceanic crust.

## Abstract (web summary)

In this study, the authors report the use of micro-electron diffractometry to identify and anisotropically refine the structure of a nanometric åkermanite inclusion in a fluid-rich diamond from South Africa.

## Introduction

Diamonds can incorporate minerals and fluids from the otherwise inaccessible deep Earth. Due to the physically robust and chemically inert nature of diamonds, such inclusions may be preserved for billions of years, from the moment of their entrapment through their eventual transport to the surface via kimberlite eruption. Diamonds thus serve as a time capsule that allows the composition and mineralogy of the mantle to be studied directly over geologic time.

Fluid-rich (cloudy/fibrous) diamonds are distinct from gem-quality samples, as they contain millions of micro- to nanometric inclusions of high-density fluids (HDFs) that have yielded important information about the composition and properties of diamond-forming media (fluids/melts). Unlike gem diamonds, which can contain large mineral inclusions (µm to mm scale), fluid-rich diamonds contain exclusively nano- to micrometric mineral inclusions. Mineral inclusions in fluid-rich diamonds can be divided into three groups: (1) primary mineral inclusions that represent mantle constituents encapsulated during diamond growth (e.g., olivine, clinopyroxene and garnet)^1^; (2) “daughter phases” that crystallize from the HDFs after diamond formation (e.g., quartz, apatite, phlogopite and carbonates)^2^; and (3) secondary phases, that form due to retrogression and/or alteration, or epigenetic phases (e.g., secondary crystallization of mineral in fractures or cracks). Primary minerals often occur as isolated inclusions but may also be found intermixed with HDF inclusions, as indicated by mineral-HDF mixing lines suggesting their mutual entrapment during diamond growth^3,4^. In contrast, daughter phases are always associated with HDF inclusions^1^.

Based on the depths at which they form, diamonds are classified as lithospheric (formation depths of ~120–200 km)^5,6^ or sublithospheric (formation depths of 300–1000 km)^7^. The maximum depth of lithospheric diamond formation is given by the depth to which the cratonic lithospheric mantle keel protrudes into the deeper convecting mantle, generally ~200–250 km. Based on the global average, lithospheric gem-quality diamonds form at 175 ± 15 km, at temperatures of 1160 ± 100 °C and pressures of 5–6 GPa^5,6^. For gem-quality diamonds, the presence of large mineral inclusions can be used to determine the pressures and temperatures, and thus the depths, at which such diamonds form, using different chemical^8^ and/or elastic^9^ geobarometric methods. Alternative methods involve the identification of particular mineral assemblages in diamonds (e.g., CaSiO_3_-breyite + Ca_2_SiO_4_-larnite + CaSi_2_O_5_-titanite in sublithospheric diamonds)^10^, or single mineral inclusions (e.g., ringwoodite^11^ and Cr-bearing clinopyroxene—the Cr-in-clinopyroxene barometer^12^) providing estimates of formation depth. However, fluid-rich diamonds do not contain mineral inclusions large enough to be readily studied using standard instrumentation (X-ray diffraction or electron microprobe), which hinders the application of geobarometric methods to such diamonds^13,14^.

Nevertheless, several studies of primary mineral microinclusions have been completed. Characterization of primary phases in fibrous diamonds has been so far based largely on electron microprobe analyses^3,14,15^. Chemical thermobarometry has been applied to different mineral microinclusions, yielding formation temperatures and pressures in good agreement with those known for gem-quality lithospheric diamonds. In such studies, geothermobarometric estimates are often supported by N-aggregation—thermochronometry. Here, the nitrogen (N)-aggregation state (e.g., %N_A_ or %N_B_)^16^ of a diamond, coupled with the rate of C- to A-center or A- to B-center aggregation (see Day et al^6^ for a detailed review on N-aggregation state), can be used to determine the model temperature at which the fluid-rich diamond resided in the mantle (Temp_res_), for a known or assumed residence time (time_res_). For gem-quality lithospheric diamonds, the global average Temp_res_ determined by N-aggregation thermochronometry are ~1150, 1180, and 1190 °C for time_res_ of 3.0, 1.0, and 0.5 Gyr, respectively^6^.

Although there is limited geothermobarometric data from primary inclusions in fluid-rich diamonds, they must form at conditions that correspond to the diamond stability field, i.e., a minimum depth of ∼120–130 km (pressure of 4.0–4.5 GPa) and temperature higher than ∼850–900 °C^17^. As evidenced by the results of geothermobarometric studies of mineral microinclusions from fluid-rich diamonds (see above), and related high-pressure and high-temperature experiments^18^, most fluid-rich diamonds are considered lithospheric^19^. However, a few diamonds with cloudy zones show typical sublithospheric features (CaSiO_3_—breyite and N contents below the FTIR detection limit—Type II diamond)^20^ but whether the cloudy appearance of such diamonds is due to H_2_O-bearing HDF inclusions, or other features (e.g., mineral inclusions and/or deformation-related features) that may also produce a cloudy appearance, has not been shown definitively.

Due to the nano- to micrometric size of mineral inclusions in fluid-rich diamonds, our understanding of such diamonds is based largely on N-aggregation thermochronometry and compositional analyses of the mineral-fluid inclusions they contain. HDF inclusions have been used to constrain the compositional range of diamond-forming media and to date fluid-rich diamonds, which are on average younger than monocrystalline gem-quality diamonds^21,22^. For example, Timmerman et al.^23^ used U–Th/He dating to show that a suite of fluid-rich diamonds from Jwaneng and DRC Congo formed much earlier than kimberlite eruption, likely between ~200 and 600 Ma. Weiss et al.^13^ suggested even older formation ages (up to 2.6 Ga) for fluid-rich diamonds from South Africa, which overlap with the typical age of lithospheric gem-quality diamonds^22^. It follows that HDF microinclusions can serve as a crucial tool to understand not only the age of fluid-rich diamonds but also the fluid-rock reactions and mantle substrates associated with their formation.

Despite the novelty of these studies, a detailed mineralogical (both structural and chemical) characterization is missing, which would greatly enhance the overall understanding of these microinclusion-bearing diamonds. A few studies have attempted to characterize the crystal structure of daughter phases crystallized from HDF inclusions. For example, apatite and high-Si mica were identified using electron diffraction (Transmission Electron Microscope—Selected Area Electron Diffraction or TEM-SAED)^2^; however, the corresponding d spacings obtained were significantly different from those expected for these two phases. In fact, mineral inclusions in fluid-rich diamonds have never been directly identified using diffraction methods^2–4,14,15,24–26^ except for the work of Smith et al^27^. These authors performed in-situ analysis of microinclusions in fluid-rich diamonds using high-intensity transmission single-crystal X-ray diffraction and identified numerous daughter phases and primary mineral inclusions. Despite the difficulties related to using in-situ single-crystal X-ray diffraction to rigorously differentiate and structurally characterize individual inclusions, the work of Smith et al.^27^ represents the most successful attempt so far to study micrometric minerals in fluid-rich diamonds.

Here, we present the definitive and direct identification of nanometric (and/or submicrometric) mineral inclusions (daughter phases) in fluid-rich diamonds by in-house single-crystal micro-electron diffractometry. Using this innovative analytical technique, we were able to identify nanometric mineral inclusions in fluid-rich diamonds that have not been previously reported in diamonds. Moreover, novel data collection and processing methods allowed the crystal structure of such nanometric inclusions to be determined, providing new fundamental information on the origin of fluid-rich diamonds.

## Results

**Fig. 1:**
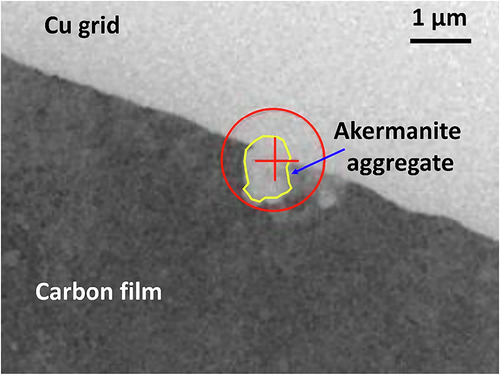
Image of an aggregate grain containing åkermanite. This grain containing analyzed åkermanite (in yellow) remaining post-combustion of the fluid-rich diamond K-09. This image was captured using the electron beam of the MED. The red circle and the cross show the exact position of the electron beam.

In order to conduct micro-electron diffraction (MED, also called 3D-electron diffraction, 3D-ED) analyses, nanoinclusions must first be released from their host diamond. To do this, diamond K-09 from the Kimberley region, South Africa, was completely combusted using a step-heating procedure at ambient pressure (with a maximum temperature of 900 °C and a total heating time between 800 and 900 °C of less than 1 h, see “Methods”). Potential effects of this combustion on mineral inclusion preservation, in addition to the type of mineral identified (e.g., primary inclusions or daughter phases), are described in detail in the Discussion. Using MED, five different grains and/or aggregates (K-09-A, K-09-B, K-09-C, K-09-D and K-09-E) were analysed (e.g., Fig. 1).

### Identification of åkermanite in diamond

Grain K-09-A represents a unprecedented finding of åkermanite (ideally Ca_2_MgSi_2_O_7_, a member of the melilite group) in diamond. This grain consists of an aggregate of åkermanite crystals (Fig. 1) measuring ~1.0 × 0.6 × 0.04 µm. Using MED, at least four different crystals were indexed in this aggregate. Plotting their orientation matrices with the OrientXplot software^28^ did not reveal any orientation relations between crystals, nor was it apparent that any of the crystals were related by twinning laws. The largest åkermanite crystal, estimated (by the percentage of indexed reflections with respect to the total number measured) to be ~0.40 × 0.24 × 0.01 µm, showed sufficient crystal quality to allow refinement of its crystal structure (refinement details are reported in Tables 1, 2). The ability to achieve an anisotropic crystal-structure refinement on a crystal of this size demonstrates the capability of the MED technique applied to geological materials.

**Table 1:** Details of electron diffraction data for åkermanite in the fluid-rich diamond K-09

Estimated crystal size (µm)	0.6 × 0.24 × 0.01
*a*, *c* (Å)	7.926(3), 5.080(3)
*V* (Å^3^)	319.1(2)
Crystal system	Tetragonal
Space group	*P*-42_1_*m*
Radiation, wavelength (Å)	Transmission electron microscope, λ = 0.0251 Å
Diffractometer	XtaLAB Synergy-ED, HyPix-ED, electron source at 200 keV
(sin θ/λ)_max_ (Å^−1^)	0.626
Reciprocal space range *hkl*	–9 ≤ h ≤ 9; –9 ≤ k ≤ 9; –6 ≤ l ≤ 6
*R*_int_	0.194
Set of read reflections	2162
Unique reflections	1456
Observed [*I* > 3σ(*I*)] reflections	740
No. of parameters	53
Refinement method	Full-matrix last-squares on *F*^2^
Structural refinement program	JANA2020 1.3.41
*R*_1_ for *I* > 2σ_*I*_	0.135
GooF	1.43
Δρ_max_, Δρ_min_ (e Å^−2^)	0.84−0.60

**Table 2:** Fractional atomic coordinates, equivalent-isotropic displacement parameters (Å^2^) and selected bond lengths (Å) for åkermanite in the fluid-rich diamond K-09

Site	x/a	y/b	z/c	^Occupancy^	^U^_iso_^*/U^eq
X	0.3336(4)	0.1666(4)	0.5079(10)	1.18(3)	0.0299(16)
T2	0.1403(5)	0.3597(5)	0.9381(13)	1.03(3)	0.0085(18)
T1	0	0	0	1.17(6)	0.018(3)
O1	0.5	0	0.183(4)	1.00	0.025(5)
O2	0.1410(9)	0.3590(9)	0.253(2)	1.00	0.021(2)*
O3	0.0807(10)	0.1825(10)	0.7898(16)	1.00	0.023(3)
**Selected bond lengths**					
X	O3	2.749(9) × 2	T1	O3	1.908(8) × 4
	O1	2.491(13)	TAV* (°^2^)		3.66
	O2	2.730(9) × 2			
	O3	2.467(9) × 2	T2	O1	1.688(8)
	O2	2.516(9)		O3	1.662(9) × 2
<X–O>		2.612		O2	1.601(13)
			<Si-O>		1.653
			TAV* (°^2^)		48.71

*after^37^; all anisotropic displacement parameters are reported in the deposited CIF file.

Minerals from the melilite group, such as åkermanite, have a general formula X_2_T1T2_2_O_7_, where X is a large mono- to divalent cation (X = Na, Ca, Sr, Ba, …), and T1 and T2 are small di- to tetravalent cations (T1 = Be, Mg, Al, Co, Zn, …; T2 = Al, Si, Ge, …). The structure can be described as layers of linked tetrahedra (T2_2_O_7_ dimers and four-coordinated T1 cations) connected by X cations accommodated in a distorted eight-coordinated antiprismatic site. Refinement of the X-site occupancy of the åkermanite crystal shows it is dominated by Ca, but the mean atomic number (m.a.n.) at this site is 23.6(6) electrons per site (e.p.s.), indicating partial occupancy by heavier cations such as Sr and/or Ba, which have larger ionic radii. Based on the average observed <X–O> bond length of 2.612 Å (significantly longer than the <X-O> distance for an X-site occupied by exclusively Ca, i.e., 2.573 Å)^29^ and on the m.a.n. At the X-site, we opted for Sr as the most likely Ca-substitute; thus, the occupancy of the X-site should be expressed as ∼Ca_1.6_Sr_0.4_. Using this X-site occupancy, and the observed <Ca–O> and <Sr–O> distances in Sr-åkermanite (2.663 Å)^30^, we calculated, for an occupancy of Ca_1.6_Sr_0.4_, an average bond length of 2.591 Å, in good agreement with the observed value. The lattice parameters and cell volume (Table 1) are larger than those for åkermanite or gehlenite [Ca_2_Al(AlSi)O_7_] and closer to those of synthetic Sr_2_MgSi_2_O_7_, i.e., *a* = 7.996(1) Å, *c* = 5.1521(9) Å, and *V* = 329.54(8) Å^3^.

The T1-site of the åkermanite has an occupancy slightly higher than unity using the scattering curve of Mg [1.17(6)], suggesting that a minor amount of a heavier cation may occupy the T1-site in addition to Mg. The observed <T1-O> distance [1.908(8) Å, Table 2] is consistent within error with the value reported for pure Ca_2_MgSi_2_O_7_ åkermanite (1.916 Å)^29^. However, the observed <T1-O> distance in Sr-åkermanite [1.942(4) Å]^30^ is significantly larger. Substituting Mg for Al^3+^ is not sufficient to satisfy the observed m.a.n. of 14.0(7) e.p.s.; in addition, substitution of some Mg for Fe^2+^ at the T1-site would lead to an even larger mean bond length than observed (i.e., 1.985 Å is the mean bond length for Fe^2+^ in tetrahedral coordination)^31^. Therefore, some additional Al^3+^ is required to produce a < T1-O> distance that accords with the observed distance. The best agreement is achieved with a T1-site occupancy of Mg_0.64_Al_0.22_Fe^2+^_0.14_, which yields a calculated mean bond length of 1.903 Å and a m.a.n. of 14.18 e.p.s.

To maintain charge compensation, an equivalent number of trivalent cations must occupy the T2 site. Hence, the corresponding T2 site occupancy would be Si_1.78_Al_0.22_. This composition yields a calculated mean bond length of 1.638 Å which is significantly shorter than the observed bond length (1.653 Å, Table 2). Moreover, the m.a.n. at the T2 site is higher than 14 e.p.s. suggesting that some Fe^2+^ may also be disordered over the T2 sites. We find a much better match with the composition of the T2 site corresponding to an occupancy of Si_1.86_Fe^2+^_0.04_Al_0.03_, which corresponds to a m.a.n. of 14.4(4) e.p.s. and a mean bond length of 1.643 Å, in closer agreement with the refined model. However, we could not find evidence in the literature for Fe^2+^ at the T2-site in åkermanite or gehlenite. Although Fe^3+^ may be ordered at the T1 site in synthetic samples^32,33^, this cation distribution would result in excess charge at the T1 site that would require compensation elsewhere. This suggests that the studied åkermanite has a 22% gehlenite component [Ca_2_Al(AlSi)O_7_].

The anomalous behavior of the observed mean bond lengths for the T1 and T2 sites could raise concerns regarding some aspects of the crystal-structure refinement, and therefore, this issue was investigated further. Calculating the T/X ratio where T/X = [(<T1-O> + 2 <T2-O>)/3 <X-O>]^34^, we obtain 0.665 that corresponds to a Δ_TAV_ (TAV_T2_–TAV_T1_; TAV = tetragonal angle variance)^35^ of ca. 45°^2^ (Fig. 3 of ref. ^34^). This is in reasonable agreement with the calculated Δ_TAV_ for our model of 48.71°^2^ (Table 2) and thus supports the reliability of our data. Moreover, using the same approach, we can exclude the presence of Na in our sample. Indeed, the Δ_TAV_ values for sodian strontian melilites are of ca. 6–7°^2^, corresponding to more distorted T1 and T2 sites (34.02 and 26.23°^2^ for T1 and T2, respectively). The anomalously long observed <T2-O> distance may depend on local configurations, as the T2 tetrahedron shares three edges with three neighboring X-sites. Occupancy of some X-sites by Sr may produce a local configuration in which the O3-O3 edge elongates, and consequently the Si-O3 distance becomes longer, leading to an eventual increase in the mean bond length. The O3-Si-O3 angle observed in our sample [106.0(7)°] is wider than that observed in synthetic åkermanite (105.62°)^29^ but narrower than that observed in Sr-åkermanite [107.2(3)°]^30^. Thus, the Δ_TAV_-T/X plot helps us to exclude the presence of detectable amounts of Ba, which would lead to negative Δ_TAV_ values [−55.85°^2^ observed for the structure of Ba_2_MgSi_2_O_7_ where O3-Si-O3 = 108.0(4)°]^36^.

Based on the considerations above, the composition of the åkermanite crystal found in diamond K-09-A can be written as follows:

Ca_1.60_Sr_0.40_(Mg_0.64_Al_0.22_Fe^2+^_0.14_)(Si_1.78_Al_0.22_)O_7_

To the best of the authors’ knowledge, a nanometric mineral released from a fluid-rich diamond has never been previously refined anisotropically by single-crystal diffraction.

### Carbonate and other inclusions

The aggregate grain K-09-B (1 µm × 0.6 µm × 0.05 µm) comprises a mixture of three different carbonates identified by MED (Table 3):

**Table 3:** Unit-cell parameters of different inclusions found in the fluid-rich diamond K-09 determined by single-crystal electron diffraction

Grain #	Unit-cell parameters
Grain **K-09-A**	
Åkermanite (tetragonal) [Ca_1.60_Sr_0.40_(Mg_0.64_Al_0.22_Fe^2+^_0.14_)(Si_1.78_Al_0.22_)O_7_]	*a* = *b* = 7.926(3) Å *c* = 5.080(3) Å *V* = 319.1(2) (Å^3^)
**Grain K-09-B**	
Norsethite (trigonal) [∼ BaMg(CO_3_)_2_]	*a* = *b* = 5.041(3) Å *c* = 17.208(12) Å *V* = 378.6(4) (Å^3^)
Monoclinic Ca-rich strontianite (monoclinic) [∼ Sr_0.75_Ca_0.25_CO_3_]	*a* = 8.190(9) Å *b* = 5.040(3) Å *c* = 6.462(12) Å *β* = 107.48(15)° *V* = 254.4(5) (Å^3^)
Nyerereite (hexagonal) [∼ (Na,K)_2_Ca(CO_3_)_2_]	*a* = *b* = 5.043(4) Å *c* = 12.911(11) Å *V* = 284.3(4) (Å^3^)
**Grain** **K-09-C**	
Sellaite (tetragonal) (MgF_2_)	*a* = *b* = 4.665(6) Å *c* = 2.999(2) Å *V* = 65.28(13) (Å^3^)
**Grain** **K-09-D**	
Anatase (tetragonal) (TiO_2_)	*a* = b = 3.814(3) Å *c* = 9.631(4) Å *V* = 140.58(14) (Å^3^)
Monoclinic Ca-rich strontianite (monoclinic) [∼ Sr_0.75_Ca_0.25_CO_3_]	*a* = 8.183(15) Å *b* = 5.031(6) Å *c* = 6.468(10) Å *β* = 107.95(17)° *V* = 253.3(7) (Å^3^)
**Grain** **K-09-E**	
Monoclinic Ca-rich strontianite (monoclinic) [∼ Sr_0.80_Ca_0.20_CO_3_]	*a* = 8.23(2) Å *b* = 5.07(2) Å *c* = 6.450(12) Å *β* = 107.8(2)° *V* = 256(1) (Å^3^)

- norsethite [BaMg(CO_3_)_2_]: previously reported in two diamonds^27^, one from the Panda kimberlite, Ekati mine (Northwest Territories, Canada) and one from Wawa metaconglomerate in the Michipicoten Greenstone Belt (Ontario, Canada);

- monoclinic Ca-rich strontianite [Sr_0.75_Ca_0.25_CO_3_]: although the unit-cell parameters of this carbonate (see Table 3) are roughly comparable with those of barytocalcite, the unit-cell volume is significantly larger. Apart from barytocalcite, we could not find any other natural analog with similar unit-cell parameters and volume. However, this mineral inclusion appears isostructural with the phase Sr_0.75_Ca_0.25_CO_3_ synthetized along the CaCO_3_-II (calcite-II) – SrCO_3_-II (strontianite-II) join^37^. This phase has never been previously reported as a mineral inclusion in diamonds, and no natural analog has been reported. Ca-rich strontianite-II was also found in two other inclusions, K-09-D and K-09-E (with variable Sr-Ca contents; see Table 3);

- nyerereite [(Na,K)_2_Ca(CO_3_)_2_]: previously reported in one lithospheric^38^ and one sublithospheric diamond^39^.

Although rare, these carbonate minerals (except for Ca-rich strontianite-II, which has never been previously reported in nature) are commonly found in carbonatites^40,41^. Norsethite, in settings outside the carbonatite association, is also found in late-stage quartz-carbonate veins^42^ and in the gangue minerals of zinc-lead-copper deposits, associated with calcite and dolomite^43^.

*-* sellaite [MgF_2_]: was identified in grain K-09-C and anatase (TiO_2_) in grain K-09-D. Sellaite and anatase can be found in calciocarbonatitic, natrocarbonatitic, and ferrocarbonatitic rocks, often in association with nyerereite^44^. Sellaite was previously observed in two different diamonds^27,45^. Anatase, as reported in Table 3, has a unit-cell volume ∼3% larger than those reported in the literature, suggesting minor Ti-substitution by other elements such as Sn.

### FTIR spectroscopy: HDF inclusions, nitrogen content and aggregation state

Visual inspection of sample K-09 revealed three distinct zones (see “Methods”). FTIR analyses of these three zones showed the following: zone a comprises gem-quality Type IaAB diamond (%N_B_ = 49–74) with no absorbance due to microinclusions; zone b comprises microinclusion-bearing fluid-rich Type IaAʹ diamond (%N_A_ = 100)^16^ and shows intense absorption peaks due to H_2_O–HOH bending (~1640–1650 cm^−1^), OH-stretching (~3400 cm^−1^), carbonates (at ~1435–1445 and 874–878 cm^−1^) and several weak peaks possibly due to quartz (~1100, 812 and 784 cm^−1^), phlogopite (~1000 cm^−1^) and apatite (~1100 cm^−1^)^1,14^; and zone c comprises gem-quality Type IaAʹ diamond (%N_A_ = 100) with no signal due to H_2_O, carbonates or silicates. Additional peaks attributed to primary silicates and/or daughter phases are described in Supplementary Material.

**Fig. 2:**
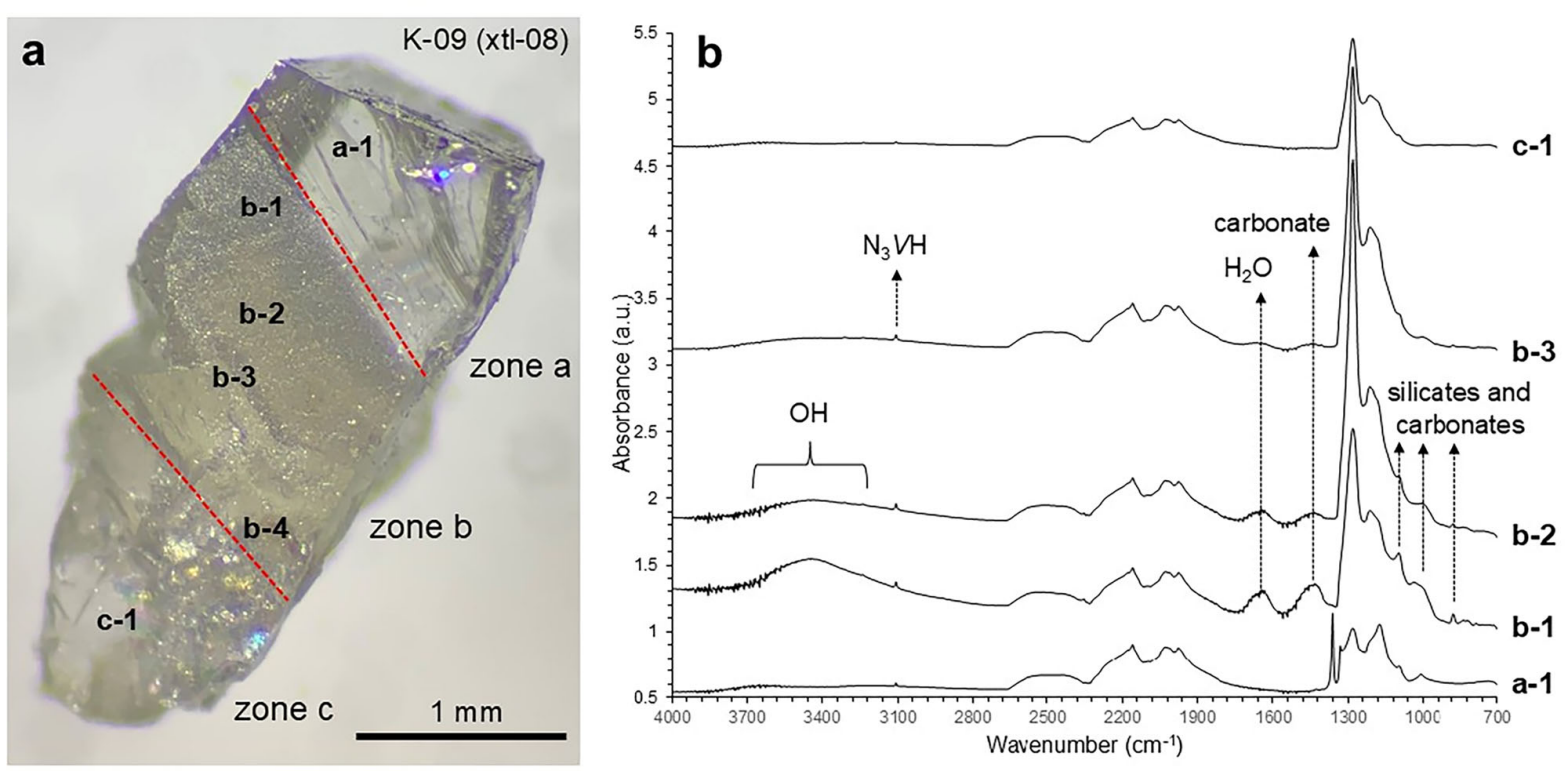
Image of each growth zone in diamond K-09 and corresponding FTIR spectra. **a** optical image of a zoned fragment of the fluid-rich diamond K-09 showing zone a (gem-quality Type IaAB), zone b (microinclusion-bearing fluid-rich Type IaAʹ) and zone c (gem-quality Type IaAʹ). **b** FTIR spectra recorded from this fragment showing peaks due to H_2_O and carbonates exclusively in the fluid-rich zone b. Spot locations for each spectrum are marked respectively in (**a**). Spectra were baselined in OMNIC^TM^.

Of the diamond fragments described above, three were identified with all three zones a–c, such that growth relationships could be evaluated (e.g., Fig. 2a). Sharp contacts are observed between zones a and b, and a gradational contact is observed between zones b and c. Zone b is markedly opaquer at the contact with zone a and becomes progressively more transparent towards zone c (Fig. 2a); the degree of opaqueness is typically correlated with the density of micro- to nanometric inclusions. FTIR traverses across zone b show a gradual decrease in the intensity of peaks due to carbonates and H_2_O towards the gem-quality zone c (Fig. 2b), indicating a higher density of fluid inclusions close to the contact between zone a and b.

Following the methods of Weiss et al.^1,46^, the position, shape and thickness-normalized intensity of bands due to OH-stretching, H_2_O bending and carbonates were obtained and used to determine the HDF type and the bulk H_2_O and carbonate contents in each zone (see Supplementary Table 1, Supplementary Fig. 1).

By comparing the width (FWHM, cm^−1^) of the OH-stretching signal to the 3200/3400 cm^−1^ absorption ratio and the position of the H–O–H (H_2_O) bending peak, Weiss et al.^46^ showed that previously defined types of HDFs can be approximately differentiated (chemical analysis is required for definitive identification of HDF composition/type). As shown in Supplementary Fig. 2, the OH-stretching and H–O–H peak characteristics mostly overlap with those of low-Mg carbonatitic to silicic HDFs, in agreement with the EMPA data from zone b as described below.

The deconvolution results and calculated Temp_res_ for seven diamond fragments are given in Table 4. In general, the total N content (N_tot_) is highly variable but is lower in zone a (116–614 at.ppm) compared to the microinclusion-bearing fluid-rich zone b (404–1577 at.ppm), and N_tot_ is less variable in zone c (770–849 at.ppm). As zones b and c, which are assumed to have formed from the same growth event, have an N-aggregation state of %N_A_ = 100, only a minimum and maximum Temp_res_ could be calculated (see “Methods” and Discussion).

**Table 4:** Selected FTIR spectroscopic and thermochronometric data from zones a, b and c from different fragments of the fluid-rich diamond K-09

fragment	zone	N_tot_	%N_A_	%N_B_	Min-max Temp_res_ (time_res_ = 2515 Myr)		Min-max Temp_res_ (time_res_ = 665 Myr)	Temp_res_				
								time_res_ 100 Myr	time_res_ 500 Myr	time_res_ 1000 Myr		time_res_ 3000 Myr
**xtl-08**					fluid-rich and gem rim (zones b + c)			gem core (zone a)				
gem(core)	a-1	574	–	69	–	–		1243	1198	1180		1152
fluid-rich	b-1	957	100	–	815–1019	841–1046		–	–	–		-
fluid-rich	b-2	1568	100	–	815–998	840–1025		–	–	–		-
fluid-rich	b-3	1341	100	–	815–1006	841–1033		–	–	–		-
gem(rim)	c-1	829	100	–	815–1025	841–1053		–	–	–		-
**xtl-01**												
gem(core)	a-1	303	–	60	–	–		1250	1205	1187		1159
gem(core)	a-2	347	–	59	–	–		1244	1200	1182		1154
fluid-rich	b-1	404	100	–	816–055	841–1085		–	–	–		-
fluid-rich	b-2	710	100	–	815–1031	841–1059		–	–	–		-
fluid-rich	b-3	1338	100	–	815–1006	841–1033		–	–	–		-
fluid-rich	b-4	1577	100	–	815–998	841–1025		–	-–	–		-
**xtl-03**												
gem(core)	a-1	614	–	74	–	–		1247	1203	1185		1157
gem(core)	a-2	116	–	49	–	–		1263	1218	1199		1170
fluid-rich	b-1	446	100	–	815–1051	841–1080		–	–	–		-
fluid-rich	b-2	990	100	–	815–1017	841–1045		–	–	–		-
fluid-rich	b-3	1019	100	–	815–1016	841–1044		–	–	–		-
**xtl-07**												
gem(core)	a-1	436	–	64	–	–		1244	1199	1181		1153
fluid-rich	b-1	1413	100	–	815-1003	841-1030		–	–	–		-
**xtl-05**												
fluid-rich	b-1	900	100	–	815–1021	841–1049		–	–	–		-
fluid-rich	b-2	1113	100	–	815–1013	841–1040		–	–	–		-
**xtl-02**												
gem(rim)	c-1	770	100	–	815-1028	841-1056		–	–	–		-
gem(rim)	c-2	849	100	–	815-1024	841-1052		–	–	–		-
**xtl-04**												
gem(core)	a-1	580	–	69	–	–		1243	1198	1180		1152
gem(core)	a-2	367	–	61	–	–		1244	1200	1182	1154	

Baseline correction and thickness normalization were done using the DiaMap_Fluid Excel spreadsheet^72^ and deconvolution of the signal in the one-phonon region was done using the Caxbd_Inherit_2024-Ib Excel spreadsheet^73^.

Model residence temperatures (Temp_res_) for zone a were calculated by standard thermochronometric methods^74^. For zone b + c, %N_A_ = 100% and thus the PQL_C_ and PQL_B_ were used to calculate the minimum and maximum Temp_res_, respectively^16^.

All temperatures are in °C. N_tot_ is expressed in at.ppm N.

### Electron microprobe analyses: silicic to low-Mg carbonatitic HDFs

**Fig. 3:**
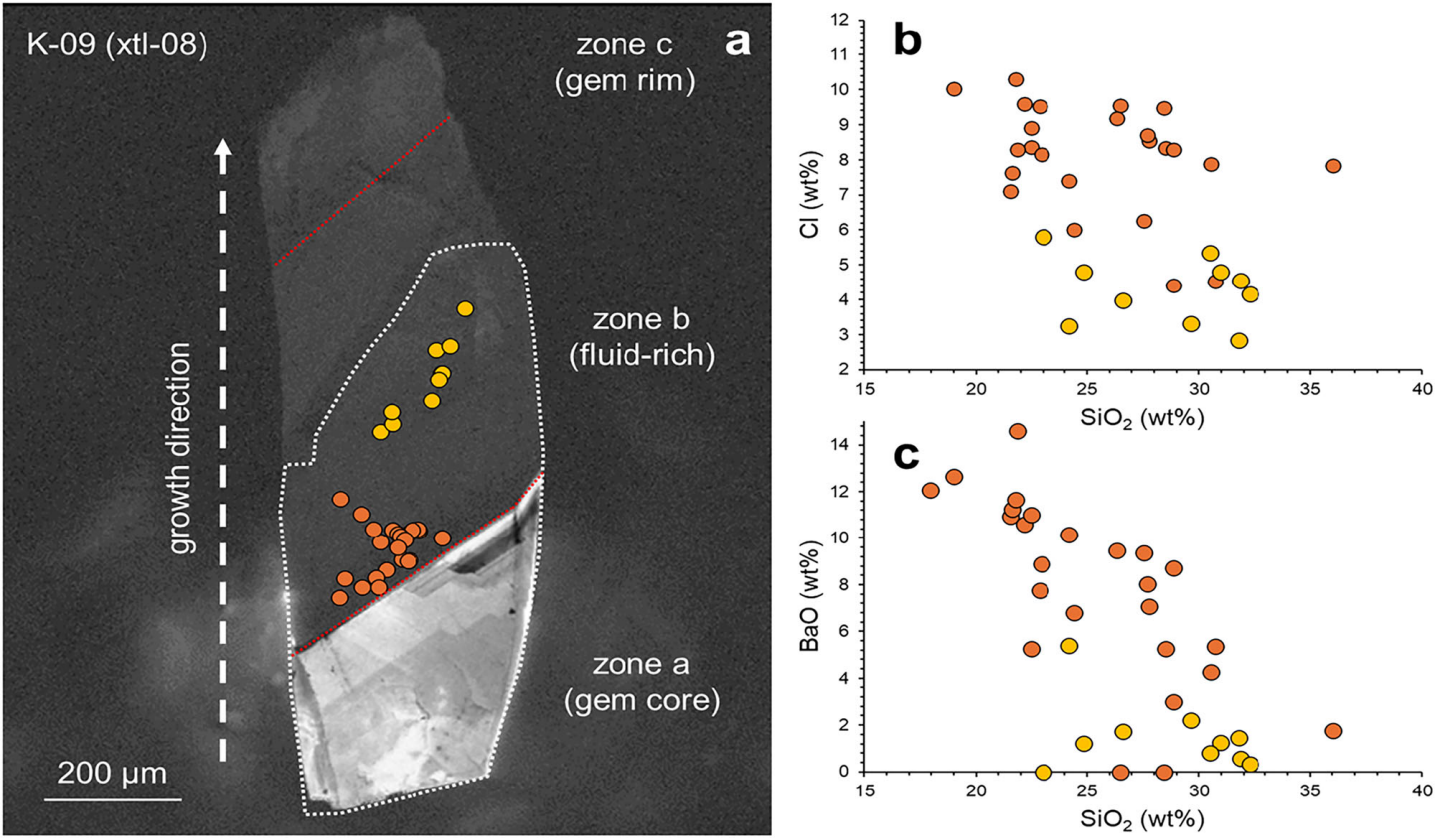
Cathodoluminescence image of polished xtl-08 (K-09) showing EMPA spots and corresponding HDF compositions. **a** CL image showing the gem-quality Type IaAB core zone a (bright region), the fluid-rich Type IaAʹ zone b and the gem-quality Type IaAʹ rim zone c. EMPA points in the darker H_2_O- and carbonate-rich region (zones b-1 and b-2 of Fig. 2) are shown as orange circles, and points in the lighter H_2_O- and carbonate-poor region (zones b-3 and b-4 of Fig. 2) are shown as light orange circles (e.g., see Supplementary Table 1). Zones a and c are indicated with red dashed lines, and the polished area is outlined with a white dashed line. EMPA data are plotted as a function of SiO_2_ vs. Cl in (**b**) and BaO (wt%) in (**c**), showing HDFs enriched in Cl and Ba in zones b-1 and b-2, respectively. Data from all individual EMPA points is given in Supplementary Data 1.

**Fig. 4:**
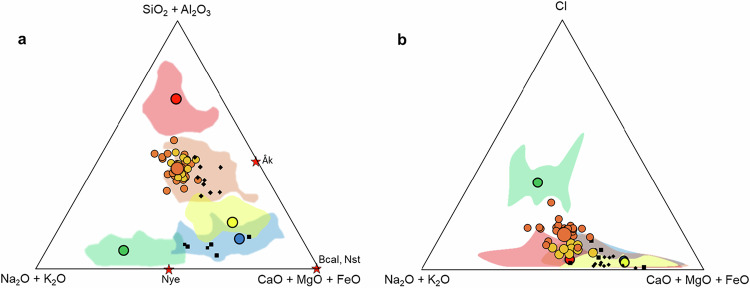
Compositional fields for the main types of HDFs plotted with the EMPA data from diamond K-09. Fields for silicic (red), low-Mg carbonatitic (yellow), high-Mg carbonatitic (blue) and saline (green) plotted in **a** SiO_2_ + Al_2_O_3_ – Na_2_O + K_2_O – CaO + MgO + FeO and **b** Cl – Na_2_O + K_2_O – CaO + MgO + FeO ternary space. The orange field represents the join between silicic and low-Mg carbonatitic compositions. The global average composition of silicic, low-Mg carbonatitic, high-Mg carbonatitic and saline fluid inclusions are shown with red, yellow, blue and green circles, respectively (modified from the dataset of Weiss et al^47^). EMPA data from K-09 (zone b) are shown with small orange circles (light orange for the zones b-3 and b-4 and darker orange for zones b−1 and b-2) and the average composition (*n* = 36) with a large orange circle. HDF compositions for other silicic to low-Mg carbonatitic (black diamonds) and high-Mg carbonatitic (black squares) fluid-rich diamonds from the Kaapvaal craton are shown for comparison (data from Weiss et al^47^). The compositions of minerals identified by MED are shown with red stars. Åk = åkermanite, Nye = nyerereite, Bcal = barytocalcite, Nst = norsethite.

The average major element content of HDF inclusions from zone b of the same crystal fragment analysed via FTIR (xtl-08, see Figs. 2, 3) is provided in Table 5. EMPA results show a silicic to low-Mg carbonatitic composition, which is consistent with the global average composition of silicic to low-Mg carbonatitic HDFs^47^, including those from the Kaapvaal craton (Fig. 4).

**Table 5:** Average composition of HDF inclusions from the fluid-rich zone of sample K-09

*n* = 36	SiO_2_	TiO_2_	Al_2_O_3_	FeO	MgO	CaO	BaO	Na_2_O	K_2_O	P_2_O_5_	Cr_2_O_3_	SO_3_	MnO	SrO	Cl*	Total
Oxide wt%	0.70	0.06	0.14	0.18	0.06	0.38	0.18	0.10	0.49	0.12	0.01	0.02	0.01	0.11	0.22	2.79
SD	0.39	0.05	0.09	0.12	0.04	0.23	0.20	0.06	0.30	0.09	0.02	0.01	0.02	0.06	0.2	1.59
	Composition normalized to 100 wt.% on a H_2_O and carbonate-free basis, with excess oxygen for chlorine															
*Oxide wt%	25.9	2.1	5.2	6.4	2.4	13.9	6.2	3.9	18.0	4.5	0.6	0.6	0.6	4.3	7.2	101.6
SD	4.5	1.9	1.3	2.6	0.9	2.5	4.6	1.5	1.9	1.8	1.1	0.5	1.1	2.2	2.6	0.6
Min.	15.5	0	2.2	0.3	0.4	7.5	0	0.3	13.9	0.6	0	0	0	0	2.8	100.6
Max.	36.0	6.2	8.0	11.9	4.2	18.6	14.6	6.7	21.8	8.4	3.8	1.4	4.4	8.9	15.9	103.6

*Average oxide wt% after normalization (number of point analyses, *n* = 36). F contents are below the detection limit. See Supplementary Data 1 for all EMPA data from individual point analyses.

However, HDFs from zone b show higher average BaO (6.3 wt%), K_2_O (18.0 wt%) and Cl (7.2 wt%) contents than are typically observed in silicic and low-Mg carbonatitic HDFs (average BaO = 0.4–0.5, K_2_O = 12.4–13.0 and Cl = 0.9–2.3)^47^. In general, analyses of the darker HDF-rich region, near the zone a-b contact (zones b-1 and b-2), show significantly higher BaO and Cl contents compared to the lighter region (zones b-3 and b-4) closer to zone c (see Fig. 3, Supplementary Data 1). No analogous trends for any other elements are observed in the darker and lighter fluid-rich zones. The abundance of BaO and SrO supports the identification of Sr-åkermanite, norsethite and monoclinic Ca-rich strontianite by MED, and the silicic to low-Mg carbonatitic composition accords with the compositions of HDFs based on FTIR data (see Supplementary Fig. 2).

### Elemental aAnalyzer – isotope ratio mass spectrometry (EA-IRMS): trends in δ13C

The results of bulk δ^13^C isotopic analyses of each zone (i.e., zones a, b and c) of sample K09 are reported in Supplementary Table 2. The gem core (zone a*)*, fluid-rich rim (zone b) and gem rim (zone c) have δ^13^C values ranging from −5.72–−6.61‰, −7.14–−7.76‰ and −7.80–−7.84‰, respectively (see Supplementary Table 2). These values are in good agreement with the previously published range for fibrous diamonds, −10.9 to −2.4‰, with a mode at −6.12 ± 1.2‰^47^. The δ^13^C of the fluid-rich rim (zone b, and in general for fibrous diamonds) is slightly more negative than the typical mantle δ^13^C value (e.g., −5 ± 2‰)^48^, and the median δ^13^C for peridotitic (−4.9‰) and eclogitic diamonds (main mode at −5.0‰ and a second mode at −11.8‰)^47,48^. The gem-quality core (zone a) overlaps the lower end of the normal mantle array^48^.

## Discussion

Before attempting to evaluate the pressure and temperature conditions at which diamond K-09 formed, it is crucial to evaluate the potential effect of diamond combustion on the preservation of the identified mineral inclusions and the possibility that such inclusions formed in the process. For the carbonate phases, this is a difficult task, as they can form at temperatures below those attained during diamond combustion (~900 °C under oxidative conditions, see “Methods”)^49^. On the other hand, no pressure—temperature stability field for åkermanite has been published. However, more robust constraints can be provided as phases with a composition comparable to the åkermanite identified in this work are typically synthesized at 1000–1350 °C^33,50,51^. Although more recent works have demonstrated that melilite-like phases can be synthesized at lower temperatures (~900 °C)^52^, prolonged (multi-hour) and high-energy ball milling (to reduce grain size and enhance mixing) are required. Even in this case, stabilization requires a dwell time of one hour, conditions that were not used during the diamond combustion procedure adopted in this work (peak temperature of ~900 °C attained for only ~1 min). Moreover, according to the temperature-composition phase diagram reported by Deer et al.^53^, the formation temperature of an åkermanite phase with a composition similar to that reported here (e.g., åkermanite 78%—gehlenite 22%) is lower than 1390 °C, just below the solidus of the åkermanite–gehlenite binary join. We can therefore state that the åkermanite inclusion found in diamond K-09 did not form due to the combustion procedure, as the maximum temperature of combustion is lower than that needed to produce this silicate.

All the collected data suggest that åkermanite is a daughter phase. The åkermanite-bearing fluid-rich zone b formed prior to the gem-quality zone c (Fig. 2) and therefore epigenetic formation of åkermanite from the kimberlite magma is not possible. Moreover, the enrichment of alkalis and incompatible elements (Sr and Ba) in åkermanite and the carbonates identified here suggests these inclusions are daughter phases crystallized from HDF inclusions after fluid-rich diamond formation. As these Ba- and Sr-rich phases are not major constituents of the metasomatized eclogitic or peridotitic substrates from which silicic-carbonatitic HDFs form^47,54,55^, it is unlikely they are primary phases encapsulated during fluid-rich diamond formation. When, where and under what conditions these daughter-phases crystallized from the silicic to low-Mg carbonatitic HDFs in diamond K-09 remains the main question.

The significantly higher N-aggregation state of the Type IaAB – core (zone a) of diamond K-09 and the sharp contact between zones a and b suggest that this diamond did not form during a single growth episode. This is also supported by the slightly different δ^13^C recorded between zones a and b. The more aggregated core (zone a) is older and may have resided at relatively higher Temp_res_ compared to the less aggregated fluid-rich zone b and the outer rim zone c, which grew during a later growth event as a rim around zone a. However, to determine the difference in Temp_res_ between the core and rim zones, time_res_ must be constrained for each zone.

Based on silicate and carbonate melt experiments in the CaO-MgO-SiO_2_-CO_2_-H_2_O system, Otto and Wyllie^56^ showed that synthesis of åkermanite requires SiO_2_ contents of 20 wt% or greater, where lower SiO_2_ content results in the formation of forsterite-, monticellite-, or periclase-bearing assemblages instead. This accords with the silicic to low-Mg carbonatitic composition of HDFs observed in zone b (average SiO_2_ = 25 wt%) and with the low-Mg and high-Mg carbonatitic HDF inclusions observed in other fluid-rich diamonds from the Kaapvaal craton^47^. Although alkali and alkaline-earth bearing daughter phases (e.g., Ba- and Sr-bearing carbonates) have been previously observed in silicic to low-Mg carbonatitic HDF inclusions^47^, the compositions analysed in zone b are enriched in BaO, SrO, K_2_O and Cl compared to the global average composition of silicic to low-Mg carbonatitic HDFs^47^. Enrichment in such elements may point to a more complex, possibly multi-source origin for the fluid-rich diamond studied here. Saline fluids, associated with seawater-altered slab-derived sources^4,15,24,57^, contain significantly higher BaO and Cl contents (global average of 8.4 and 32.6 wt%, respectively)^47^. Formation of silicic to low-Mg-carbonatitic fluids has been previously attributed to a hydrated and carbonated eclogitic source^58,59^. It follows that percolation of slab-derived hydrous carbonated fluids (i.e., saline) into an eclogitic substrate may produce a silicic to low-Mg carbonatitic composition enriched in Ba, Cl and K as observed here. The involvement of a saline fluid is supported by the observation of nyerereite, the shift in the average composition of HDFs towards the saline end-member (Fig. 4) and the partial overlap with the saline field (Supplementary Fig. 2) where data collected from the Ba- and Cl-rich region (zones b-1 and b-2) accords with that of NaCl solvated water. These findings accord with the composition of HDFs in fluid-rich diamonds from the Kaapvaal Craton reported in the literature, the majority of which (~80%) are saline^47^. Although most of these samples have been assigned to peridotitic parageneses, minor saline fluid-rich diamonds from the Kaapvaal Craton with eclogitic micromineral inclusions have been previously described^4,14,25,57^. Interestingly, the BaO content of HDFs in zones b-1 and b-2 is higher (up to 14.6 wt%) compared to other saline fluid-rich diamonds from this area (< 8 wt%)^47^. On the other hand, Cl contents of HDFs are lower (~4-16 wt%) compared to other saline fluid-rich eclogitic diamonds from the Kaapvaal Craton with Cl contents ranging from ~30 to 40 wt%^14,25,57^. Several authors^54,60,61^ observed a correlation between the BaO, Cl and K_2_O contents of HDFs in fibrous diamonds, implying preferential partitioning of Ba into the saline fluid component. It is likely that the general decrease in BaO and Cl away from the core-rim boundary (see Fig. 3b, c) results from an increasing degree of melting, which causes a progressive dilution of strongly incompatible elements from the initial pristine melt. Such a trend seems to be uncommon in literature, as most of the published data on fluid-rich diamonds show spatially homogenous HDF compositions. So far, only nine fluid-rich diamonds (~ 2% of the studied fluid-rich diamonds) show a spatial trend in the HDF composition, as is observed for K-09^15,54,61^. These few other diamonds with K- and Cl-rich silicic to low-Mg carbonatitic HDF compositions (similar to HDFs in K0-9) have been observed and interpreted to form from the interaction between saline fluids and eclogitic lithologies^15,54,61^.

Li et al.^62^ suggest that altered oceanic crust (AOC) is a better candidate than carbonate sediments to reintroduce C into the deeper portions of the mantle. AOC has a C isotopic composition that partially overlaps with the normal mantle range, but can reach more negative values, down to −24‰^62^. The δ^13^C ratio of the fluid-rich rim (zone b) of K-09 can be explained by invoking AOC as a source component of the diamond-forming medium. As the fluid-rich and gem-quality rim regions (zones b and c) grew from a single diamond-forming event, equilibrium fractionation during growth of the rim may explain the minor difference in δ^13^C (i.e., 0.37‰, on average) of zone b and zone c.

Here, we propose two possible scenarios for åkermanite formation, which crystallized from its parental HDF due to decreasing pressure associated with: 1) exhumation and depressurization of the diamond prior to kimberlite eruption, to shallower regions in the lithospheric mantle; or 2) kimberlite eruption, where temperatures sufficiently high (up to 1300–1400 °C) would be maintained during eruption and depressurization to allow åkermanite crystallization. Scenario 2 is the simplest explanation; however, scenario 1 cannot be ruled out, and associated evidence for this scenario is described below.

The oldest diamonds from the Kaapvaal Craton, particularly those from the Kimberley region, have been related to multiple diamond-forming episodes during the Archean (~2.9 Ga)^63^ and the Proterozoic (1.2–1.5 Ga)^63^. It is likely that formation of the highly aggregated Type IaAB gem core, which requires significantly higher Temp_res_ and/or time_res_, is associated with Archean subduction related to the assembly of the proto-Kaapvaal craton. Formation of the silicic to low-Mg carbonatitic HDFs, and subsequent crystallization of the fluid-rich rim of diamond K-09, is likely associated with later Proterozoic formation of eclogitic diamonds. This diamond-forming event is likely related to silico-carbonatitic metasomatism in the Kaapvaal cratonic lithospheric mantle, which occurred between 2600 and 750 Ma following craton stabilization^13^. In particular, the Namaqua-Natal Orogeny (which started around 1200 to 1100 Ma)^64^ can account for the recycling of altered oceanic crust required for the formation of carbonated eclogites and the saline fluids that may account for high K_2_O, SrO, Cl and BaO contents of HDFs, as well as the δ^13^C composition of the fluid-rich zone of diamond K-09.

**Fig. 5:**
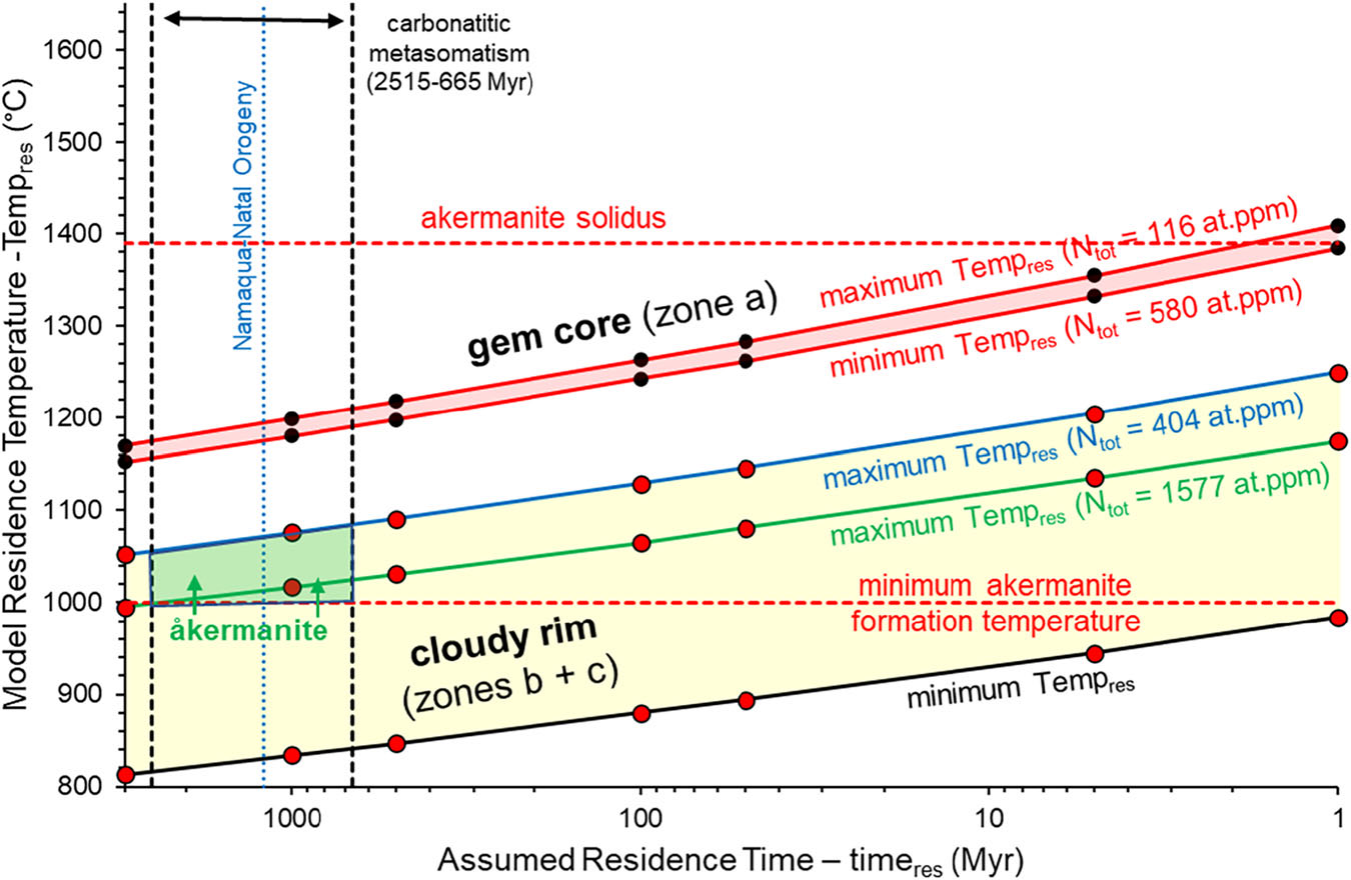
Model residence temperature (°C) plotted as a function of assumed residence time (Myr) for each growth zone of diamond K-09. Minimum and maximum Temp_res_ are calculated (for time_res_ of 3000 to 1 Myr) for zones b + c (Type IaAʹ) using the PQL_C_ and PQL_B_, and for zone a (Type IaAB) using the minimum and maximum N_tot_ (see “Methods”). Assuming carbonate metasomatism of the Kaapvaal lithospheric mantle occurred between 2600 and 750 Ma and kimberlite eruption occurred at ~85 Ma, a time_res_ for the fluid-rich zones b + c of 2515 to 665 Myr is predicted and shown with vertical black dashed lines. Assuming scenario 1, the formation temperature of the fluid-rich (åkermanite-bearing) zone can be constrained to temperatures between ~1000–1085 °C (green field), where the minimum temperature is derived from the presence of åkermanite (red dashed line). The timing of the Namaqua-Natal Orogeny (1.2 Ga), likely associated with subduction-related eclogitic diamond formation, is shown with a blue dashed line.

Considering the eruption age of the De Beers Pool kimberlites (85 ± 5 Ma)^65^ and thus a time_res_ for zones b and c of 2515–665 Myr, a minimum Temp_res_ of 815–840 °C (determined using the PQL_C_, see “Methods”) and a maximum Temp_res_ of ~1000–1085 °C (determined using the PQL_B_ and an N_tot_ = 404 at.ppm, see Table 4) can be calculated (Fig. 5). For the Type IaAB core (zone a), an assumed time_res_ of 2.9 Ga corresponds to Temp_res_ of ~1145–1185 °C (Fig. 5) assuming an average N-aggregation state of 63% N_B_. Therefore, differences in N aggregation state between the core and the rim cannot be solely explained by invoking differences in time_res_. For example, if a time_res_ of 1.2 Gyr is assumed (timing of Namaqua-Natal Orogeny and other eclogitic diamonds from the Kaapvaal) for the fluid-rich rim, a maximum Temp_res_ of ~1010–1070 °C is calculated and therefore a temperature decrease of at least ~75–175 °C (Fig. 5) is required after formation of the core zone and prior to formation of the fluid-rich rim. Even if a normal mantle cooling rate is considered (~50 °C/Gyr)^66^, some degree of upward movement of the core (zone a) would likely be required to ensure Temp_res_ is sufficiently low to inhibit the formation of B-centers in the 100% N_A_ fluid-rich rim. If a maximum Temp_res_ difference of 125 °C (see potential range above) is assumed, the fluid-rich rim would have had to form significantly later (assumed time_res_ < ~1.0 Gyr) such that mantle cooling would be sufficient to explain the observed difference in N-aggregation states (Temp_res_) in the core and rim zones. However, formation of the fluid-rich rim at <1.0 Ga is close to the upper limit of carbonatitic metasomatism (750 Ma) proposed by Weiss et al.^13^ and later than the other eclogitic diamond-forming events (e.g., 1.2–1.5 Ga). Therefore, it is likely that diamond K-09 was exhumed to a relatively cooler environment prior to the formation of the fluid-rich rim.

In scenario 1, movement of the core (zone a) to shallower environments may be only the initial phase of a prolonged and polyphasic exhumation history. Similar processes are suggested for gem-quality diamonds from the same locality (Kimberley region) by Nimis et al^67^. Using elastic (thermo)barometric data from touching and non-touching mineral inclusions, these authors show that such diamonds were transported tens of kilometers upwards during multi-stage mantle uplift associated with Archean tectonism. Subsequent pressure decreases, after formation of the fluid-rich diamonds, at temperatures ≥ 1000 °C (see explanation above), may explain åkermanite crystallization from parental HDFs. Åkermanite formation, related to exhumation and subsequent depressurization of HDF inclusions, must have occurred at temperatures ≥1000 °C, thus defining the minimum diamond formation temperature, and consequently, the minimum formation pressure of 4.6 GPa and depth of 140 km (assuming a surface heat flow of 40 mWm^−2^)^68^. Although the maximum Temp_res_ for the fluid-rich rim (~1000–1085 °C) may be different than the formation temperature, it is unlikely that the 100% N_A_ zones b + c formed at temperatures >1175 °C as detectable quantities of B-centers would form rapidly (< 15 Myr) at such temperatures.

In scenario 2, the rim of diamond K-09 (zones b and c) formed much later than in scenario 1, associated with the Mesozoic saline metasomatism, closely related to the Cretaceous Kaapvaal kimberlite eruptions^13^. The N-aggregation state of fluid-rich diamond K-09 matches the other diamonds with saline inclusions reported for the Kaapvaal Craton^4^. In this scenario, mantle cooling rates would be sufficient to explain the differences in the N-aggregation states of zone a and zone b + c.

Despite uncertainties related to the pressures and temperatures at which the various carbonates and åkermanite formed, our results clearly show that MED analysis of fluid-rich diamonds is a viable method by which a complete mineralogical (compositional and structural) characterization of fluid-rich diamond formation can be obtained. As described above, the majority of previous related work has relied solely on chemical (electron microprobe) and spectroscopic (FTIR) analyses of HDF inclusions. However, such analyses may suffer from mixed-phase sampling (e.g., bulk sampling of multiple minerals, minerals and fluids, or intergrown/exsolved phases), making rigorous mineral identification difficult due to potentially inaccurate crystal chemical data. In this regard, MED is advantageous as it allows minerals with similar or identical compositions to be differentiated based on crystallographic data. For example, differentiation of polymorphs [e.g., (Mg, Fe)_2_SiO_4_ - olivine, wadsleyite and ringwoodite] requires structural data and has provided crucial information about the *P*/*T* conditions of gem-quality diamond formation and the related retrograde (or prograde) history of inclusion assemblages in previous studies^69^. Detailed structural data, e.g., site occupancies, bond lengths and unit-cell parameters, can provide fundamental information about the valence state of elements and thus the redox conditions of diamond formation. Moreover, accurate structural formulae (as opposed to empirical chemical formulae obtained from microprobe analysis) are often required to reliably determine (theoretically or experimentally) the *P*/*T* stability of mineral inclusions and thus constrain the formation conditions of the host diamonds. This study demonstrates that MED enables the extraction of crucial mineralogical information from fluid-rich diamonds that were missing, as previous studies relied primarily on chemical data, thereby opening new research directions for understanding the genesis of these unique diamonds.

## Methods

### Sample K-09

Diamond K-09 is from the Kimberley region of South Africa, but no specific information regarding the pipe locality is available, and no previous work has been conducted on this sample. In preparation for MED analysis, the sample was combusted at high temperature (see next section), leaving behind micro- and nanometric mineral phases derived from microinclusions. As the original sample was relatively large, it had to be crushed such that fragments were preserved for spectroscopic, chemical and isotopic work. FTIR analyses revealed that sample K-09 contained zones of fluid-rich and gem-quality diamond (see Results). In an effort to maximize the yield of micro- and nanometric mineral inclusions during combustion, a fragment that consisted exclusively of microinclusion-bearing fluid-rich diamond was selected for combustion.

### Sample preparation for single-crystal electron micro-diffraction (MED) analysis

Due to the high electron-matter interaction (i.e., elastic scattering from electrostatic potential) coupled with a small beam size (<1 µm), the penetration depth of the electron beam produced by the single-crystal electron diffractometer used in this work is limited. Therefore, mineral inclusions need to be released from the fluid-rich diamond host before they can be analysed by MED. To do this, a fragment of sample K-09 (∼4 × 3 mm and 24 mg) was placed in a clean platinum crucible and heated up to 900 °C at 5 °C/min^−1^ (total heating time of 3 h). After, the sample was cooled down from 900 °C to 400 °C in 3 h, and from 400 °C to room temperature over an additional 3 h. The entire procedure was carried out in an atmosphere at room pressure. This heating-cooling procedure was sufficiently long (~9 h) to completely combust 100% of sample K-09, leaving behind only a small amount of residual powder (containing micro- and nanometric mineral inclusions). This material was recovered using isopropyl alcohol and a micropipette and then deposited on a TEM copper grid (200 mesh, 3.05 mm diameter with a pure carbon film) in preparation for MED analysis.

### Single-crystal electron micro-diffraction analysis

A XtaLAB Synergy-ED – Electron Diffractometer, located at the Rigaku Europe headquarters in Neu-Isenburg (Germany), was used in this study. This instrument is not a TEM as it is dedicated to single-crystal electron diffraction using the continuous rotation method, combining hardware and software by JEOL and Rigaku. The MED uses a JEOL 200 kV electron source (LaB₆ electron gun, electron energy up to 200 keV) with column and beam optics optimized for electron diffraction purposes. For these reasons, a wavelength λ = 0.0251 Å was used during the experiments. The MED is equipped with a Rigaku HyPix-ED detector, optimized for counting electrons under operation in the 3D-micro-ED experimental setup, and a sample stage allowing x, y, z sample alignment and rotation about a single axis. A total of 320 diffraction images of 0.25° width were collected in continuous (cRED) mode and shutterless operation using a 10 μm condenser aperture. The camera length was set at 500 mm, ensuring a maximum resolution of 0.42 Å. The electron diffractometer was controlled by the CrysAlisPro software (Rigaku). Data collection and reduction were carried out using CrysAlis Pro (version 1.171.43.90a, Rigaku OD)^70^. In this work, single and complete data collections were carried out in only two to four minutes.

### Crystal structure refinement strategy

For one of the six nanometric mineral inclusions of åkermanite, data was collected of sufficient quality and completeness (up to 83%) such that the crystal structure could be refined. The aggregate containing the crystal of åkermanite in Fig. 1 was ~1 × 0.6 × 0.04 µm, with the åkermanite crystal estimated to be ~0.4 × 0.24 × 0.01 µm. The unit-cell parameters are consistent with a tetragonal primitive cell corresponding to åkermanite, but with a significantly larger cell-volume, 319.1(2) Å^3^ compared to synthetic åkermanite [307.35(5) Å^3^]^29^. The crystal structure was kinematically refined using SHELX starting from the atom coordinates of Yang et al^29^. The X- and T1-site occupancies were refined using the scattering curves of neutral Ca and Mg. An extinction correction was also applied. The model was refined to R1 = 16.71%, but only the X-site could be refined with an anisotropic displacement parameter. Nevertheless, the geometry is in good agreement with that observed for åkermanite. Dynamical refinements were performed starting from the kinematic model using Jana2020^71^ and a new version of the Dyngo program^72^. Refinement converged to R [F^2^ > 2σ(F^2^)] = 13.5 %, significantly lower than the kinematic model. In this dynamic refinement, all atoms except for oxygen at O2 were refined with anisotropic displacement parameters and without extinction correction. All attempts to model O2 with anisotropic displacement parameters resulted in negative values for the U_33_ component. The ratio between observed reflections and the number of parameters was already > 5. The dynamic refinement results are reported in Tables 1 and 2 and details regarding the nano-inclusions identified in sample K-09 are reported in Table 3.

### FTIR spectroscopy

Fourier-transform infrared spectroscopic (FTIR) analyses were performed using a Thermo-Fisher Nicolet iN10 InfraRed microscope equipped with a KBr beam splitter and an LN-cooled MCT detector (Department of Geosciences, University of Padova - Italy). The spectra were collected at an operating resolution of 4 cm^–1^ over the range 6000–700 cm^–1^ by averaging 64 scans with a scan time of 22.4 s. Sample fragments were cleaned in a sonic bath with acetone and mounted on a BaF_2_ background window. In some spectra, regions with slightly more noise observed at 3975–3540 cm^–1^ and 1565–1430 cm^–1^ are due to atmospheric contamination by H_2_O vapor.

Visual inspection of sample K-09 revealed a transparent gem-quality core (zone a), a cloudy microinclusion-bearing rim (zone b) and an outer gem-quality rim (zone c), which were later confirmed via FTIR spectroscopy (see Results). Seven fragments that contained two or more zones were selected for FTIR analysis (Supplementary Table 1). Raw spectra were thickness-normalized and baseline corrected using the DiaMap_Fluid Excel spreadsheet^73^. Peaks due to OH-stretching, HOH-bending and carbonates were also fit using the DiaMap_Fluid Excel spreadsheet. The HOH-bending position, FWHM and 3200/3400 cm^−1^ absorption ratio of the OH-stretching peak were used to determine the HDF type following the methods of Weiss et al^46^. H_2_O and carbonate (dolomite and calcite) concentrations (wt.ppm) were calculated using the OH-stretching peak and the major carbonate peak (1430–1445 cm^−1^), respectively, following the methods of Weiss et al^46^. Deconvolution of the signal in the one-phonon region was done using the DiaMap_Fluid^72^ and Caxbd_Inherit_2024-Ib Excel spreadsheets^74^ to determine the concentration of N-defects and the N-aggregation state. For gem-quality zones (zone a) with N-aggregation states of 49–74 %N_B_ (see Day et al.^16^ for terminology), Temp_res_ for a series of assumed time_res_, were calculated using standard thermochronometric methods^16,75^. Other gem-quality zones (zone c) and all fluid-rich zones (zone b) have an N-aggregation state of 100 %N_A,_ and thus only a minimum and maximum Temp_res_ (for a series of assumed time_res_) could be calculated. This was done using the practical quantitation limit (PQL) of C- and B-centers (PQL_C_ = 2.50 ± 0.20 at.ppm; PQL_B_ = 7.94 ± 0.8) as described by Day et al^16^. Here, the PQL_C_ or PQL_B_ represents the minimum amount of C- or B-centers that produce a signal sufficiently intense such that it can be accurately deconvoluted via least-squares fitting. It follows that for a diamond with exclusively A-centers (i.e., 100 %N_A_), C- or B-center contents up to 2.50 or 7.94 at.ppm, respectively, will likely not be detected during standard deconvolution. Using these values, a minimum and maximum Temp_res_ for 100 %N_A_ diamonds can be calculated^16^.

### Electron Microprobe Analysis (EMPA)

Major element composition of microinclusions was determined using a JEOL JXA-8230 electron probe microanalyzer (EPMA). Prior to analysis, the diamond fragment was polished, cleaned using an equal volume mixture of 29 N HF and 16 N HNO_3_ to remove any surficial contaminants (including exposed microinclusions)_,_ and carbon-coated. Individual shallow subsurface inclusions were analyzed for 100 live-seconds using an accelerating voltage of 15 kV, a beam current of 15 nA, and a magnification of > 5000×. Data correction was performed using the ZAF procedure provided by JEOL. Calibration was carried out using SPI natural and synthetic mineral standards. Oxygen was calculated by stoichiometry of the major oxides. The concentrations of all oxides and chlorine totaled between 1–7 wt%, with the ZAF matrix correction assuming the remainder was carbon from the diamond. All oxide and chlorine concentrations were normalized to 100 wt% on a carbon-free and volatile-free basis (where Cl is present, excess calculated oxygen leads to a normalized total of more than 100%). Cathodoluminescence (CL) imaging was performed using the panchromatic CL detector of the EPMA, with an acceleration voltage of 15 kV, an average collection time of ~20 min, and a beam current of 190 pA.

### δ13C data: Elemental Analyzer – Isotope Ratio Mass Spectrometry (EA-IRMS)

Several fragments of diamond K-09 were selected for EA-IRMS analysis, fragments from the gem core (zone a), fluid-rich rim (zone b) and gem rim (zone c) were verified by FTIR spectroscopy. After cleaning, 25 to 48 micrograms of diamond from each zone were loaded in silver capsules and analysed at the University of Padova (Department of Geosciences), using an IRMS (Delta V Advantage) equipped with a Flash HT - Elemental Analyzer and a ConFlo IV interface. Fragments of sample K-09 were measured together with two international standards (IAEA CH-6, δ^13^C = −10.45‰ and IAEA CH-7, δ^13^C = −32.15‰) and one in-house standard (which has a standard deviation lower than 0.19‰). Blank capsules were also run in each sequence to correct the sample values for any contamination. To check for matrix-dependant bias, mixed-habit (asteriated) diamonds from Marange (Zimbabwe) were measured. These diamonds have extremely homogenous δ^13^C compositions (−8.53 ± 0.67‰ based on 251 SIMS spot analyses) and no variation amongst cuboid and octahedral sectors was detected^75^. Our results are within 2std of the average δ^13^C reported by Smit et al^76^.

## Supplementary information

Supplementary Information

Description of Additional Supplementary Files

Supplementary Data 1

Transparent Peer Review file

## Data Availability

The data supporting the findings of this study are provided in the main text and Supplementary Information.
