# Supplementary material for: Nanometric mineral inclusions from a fluid-rich diamond: identification, structure, and implications for deep Earth
Source: Nat Commun. 2026 Jun 23;17:7915. doi: 10.1038/s41467-026-74619-3 (PMC13443600; doi:10.1038/s41467-026-74619-3)
Supplement: Supplementary file 1 [file 41467_2026_74619_MOESM1_ESM.pdf]

Supplementary Material

for

Nanometric mineral inclusions from a fluid-rich diamond: identification, structure, and implications for deep Earth

Authors

Yanjuan Wang[1*,2], Fabrizio Nestola[2*], Fernando Cámara[3], Maxwell C. Day[2], Francesca Innocenzi[2], Matteo Ardit[2], Robert W. Luth[4], D. Graham Pearson[4], Yaakov Weiss[5], Suzette Timmerman[6], Martha G. Pamato[2], Davide Novella[2], Nicola Rotiroti[3], Jürgen Grässlin[7], Christian J. Schürmann[7], Anna Barbaro[2], Kai Qu[8,9], Claudia Agnini[2] & Zengqian Hou[1]

Affiliations

[1]State Key Laboratory of Deep Earth and Mineral Exploration, Chinese Academy of Geological Sciences, 100037, Beijing, China
[2]Dipartimento di Geoscienze, Università degli Studi di Padova, Via Gradenigo 6, I-35131 Padova, Italy.
[3]Dipartimento di Scienze della Terra, Università degli Studi di Milano, Via Luigi Mangiagalli 34, 20133, Milan, Italy
[4]Department of Earth and Atmospheric Sciences, University of Alberta, 116 St & 85 Ave, Edmonton, AB, T6G 2R3, Canada.
[5]Institute of Earth Sciences, The Hebrew University of Jerusalem, Jerusalem 91904, Israel
[6]Institute of Geological Sciences, University of Bern, Baltzerstrasse 1+3, 3012 Bern, Switzerland.
[7]Rigaku Europe SE, Hugenottenallee 167, 63263 Neu-Isenburg, Germany
[8]Tianjin Centre, China Geological Survey, 300170, Tianjin, China
[9] School of Earth Sciences and Engineering, Nanjing University, 210023, Nanjing, China

**Corresponding authors.*
E-mail: fabrizio.nestola@unipd.it (orcid: 0000-0002-4875-5125)
E-mail: wang.yanjuan@foxmail.com (orcid: 0000-0002-6985-2012)

FTIR data recorded from the fluid-rich zone of diamond K-09

In general, the H_2O content is highest at the contact between zone a and b (i.e., opaque zone b-1 in Fig. 2a), with average H_2O contents of ~240-300 wt.ppm, and decreases towards zone c where the average H_2O content of the lighter coloured fluid-rich zones (i.e., zones b-2 and b-3 in Fig. 2a) ranges from ~90 to 110 wt.ppm (see Supplementary Figure 1a and Supplementary Table 1). Akin to H_2O, the concentration of carbonates is highest at the zone a-b contact and decreases towards zone c (see Supplementary Figure 1b and Supplementary Table 1). In most cases, the major carbonate peak occurs at ~1440-1445 cm^{-1} with a minor peak at ~875-880 cm^{-1} which is typically attributed to a dolomitic - $CaMg(CO_3)_2$ composition (e.g., Weiss et al.[1]). However, MED did not reveal the presence of pure dolomite (or calcite) and so variability in the major and minor carbonate absorption peaks is likely due to relative concentrations of the other carbonates (norsethite, monoclinic strontianite and nyerereite) sampled by FTIR.

Apart from broad absorption due to H_2O and carbonates in HDFs, additional peaks observed at 930, 875-880, 834 and 819 cm^{-1} overlap with those for other primary silicates observed in fluid-rich diamonds (e.g., olivine and garnet) but also partially with some daughter phases (e.g., apatite and phlogopite). Consequently, the possible presence of minor amounts of such primary silicates cannot be ruled out as spectra could only be collected down to 700 cm^{-1} and thus the lower wavenumber peaks of olivine, garnet and pyroxene could not be used to confirm their presence. A relatively weak peak at 3107 cm^{-1} due to N_3VH defects[2,3] is observed in all spectra but no intensity correlations with particular zones were observed.

Supplementary Table 1. FTIR data for H_2O and carbonates collected from the fluid-rich zone of diamond K-09

	Water						Carbonate		
**sample**	**OH peak position (cm^{-1})**	**OH FWHM (cm^{-1})**	**OH 3200/3400 cm^{-1} ratio**	**HOH bending position (cm^{-1})**	**H_2O conc. (87) (wt.ppm)**	**H_2O conc. (109) (wt.ppm)**	**carb. peak position (cm^{-1})**	**carb. phase**	**carb. conc. (wt.ppm)**
dark H_2O- and carbonate-rich zones									
xtl8 zone b-1	3445	383	0.4753	1646	277	221	1445	dolomite	214
xtl1 zone b-1	3441	368	0.4112	1646	180	144	1444	dolomite	152
xtl1 zone b-2	3439	385	0.4872	1644	282	225	1443	dolomite	255
xtl3 zone b-1	3440	429	0.5881	1642	267	213	1441	dolomite	157
xtl3 zone b-2	3416	448	0.6115	1640	281	224	1439	dolomite	159
xtl5 zone b-1	3452	355	0.4209	1644	353	282	1444	dolomite	297
xtl7 zone b-1	3421	372	0.4684	1647	466	372	1444	dolomite	373
light H_2O- and carbonate-poor zones									
xtl8 zone b-2	3418	436	0.5810	1642	128	102	1435	calcite	109
xtl8 zone b-3	3411	498	0.7228	1640	54	43	1440	dolomite	109
xtl1 zone b-3	3418	400	0.5400	1645	143	114	1435	calcite	90
xtl1 zone b-4	3418	493	0.6708	1642	161	129	1431	calcite	100
xtl3 zone b-3	3410	512	0.6991	1645	87	69	1440	dolomite	44
xtl5 zone b-2	3451	370	0.5219	1648	80	63	1440	dolomite	49

Spectra were thickness normalized, baseline corrected and peaks due to OH-stretching, HOH-bending and carbonates were fit with the DiaMap_Fluid Excel spreadsheet[4].

H_2O contents (wt.ppm) were calculated using the H_2O absorptivities of $\varepsilon^{3420\ cm^{-1}}$ = 87 AU·l/mol·cm and $\varepsilon^{3404\ cm^{-1}}$ = 109 AU·l/mol·cm, respectively[5,6], and with a 9% correction applied for the effect of salinity[1].

Carbonates were identified based on the position of the major (1430-1445 cm^{-1}) and minor peaks (875-880 cm^{-1}) and concentrations were calculated following the methods described by Weiss et al.[1].

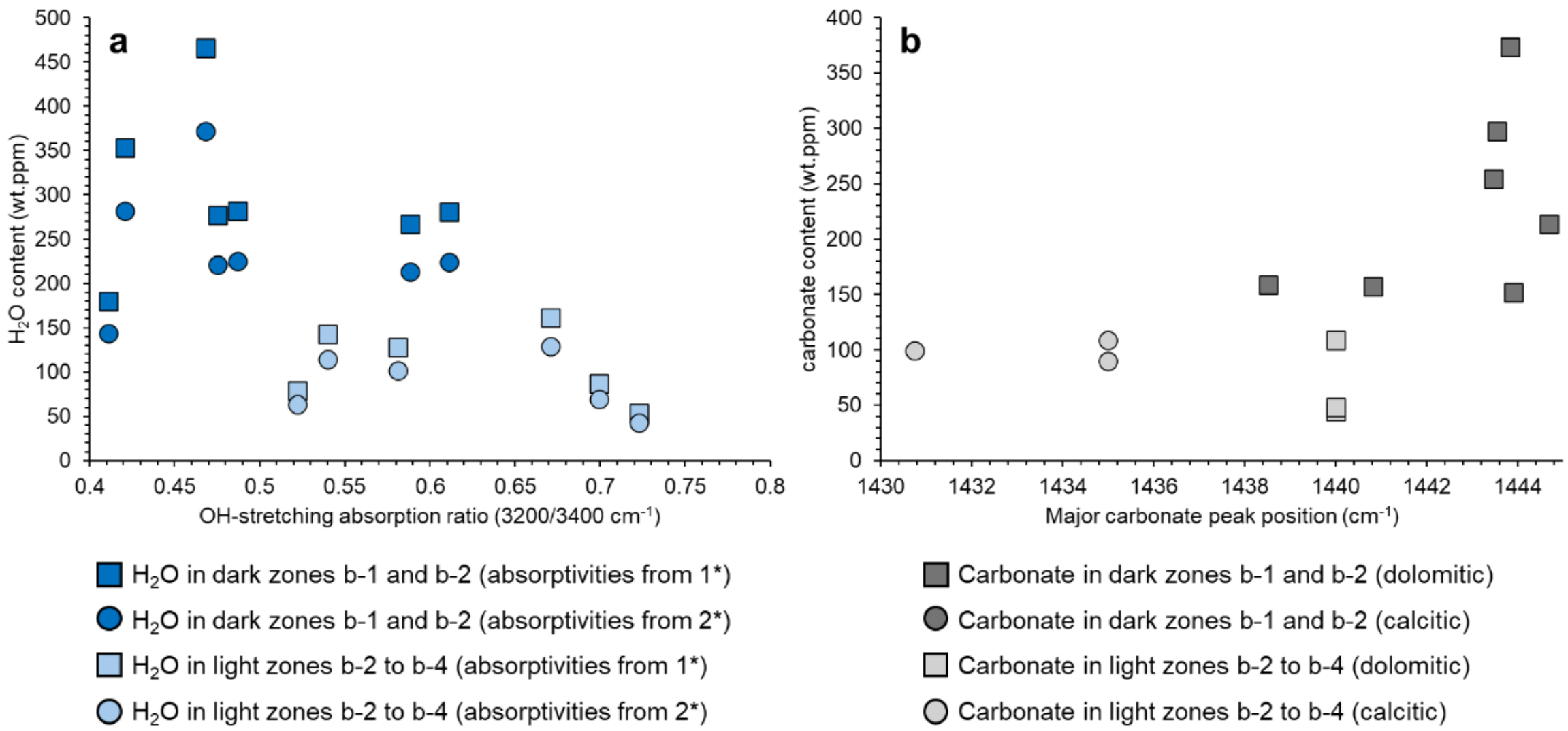

Supplementary Figure 1. a) Bulk H_2O content (wt.ppm) in diamond K-09 plotted as a function of OH-stretching absorption ratio (3200/3400 cm^{-1}). Dark blue symbols represent data collected from fluid-rich zones near the zone a-b contact (e.g., zone b-1) and light blue symbols represent other data collected from areas relatively closer to the gem Type IaA′ zone[7] (e.g., zones b-3-b-4). Blue squares and circles represent H_2O contents calculated using the H_2O absorptivities of $\varepsilon^{3420\ cm^{-1}}$ = 87 AU·l/mol·cm (1* Thompson[5]) and $\varepsilon^{3404\ cm^{-1}}$ = 109 AU·l/mol·cm, respectively (2* Venyaminov and Prendergast[6]), and with a 9% correction applied for the effect of salinity[1]. **b**) carbonate content (wt.ppm) plotted as a function of the corresponding carbonate peak position (cm^{-1}). Dark grey symbols represent data collected from fluid-rich zones near the zone a-b contact (e.g., zone b-1) and light grey symbols represent other data collected from areas relatively closer to the gem Type IaA′ zone (e.g., zones b-3-b-4). Grey squares and circles represent dolomitic and calcitic compositions, respectively. Dolomite and calcite concentrations were calculated using peak heights following the methods of Weiss et al.[1].

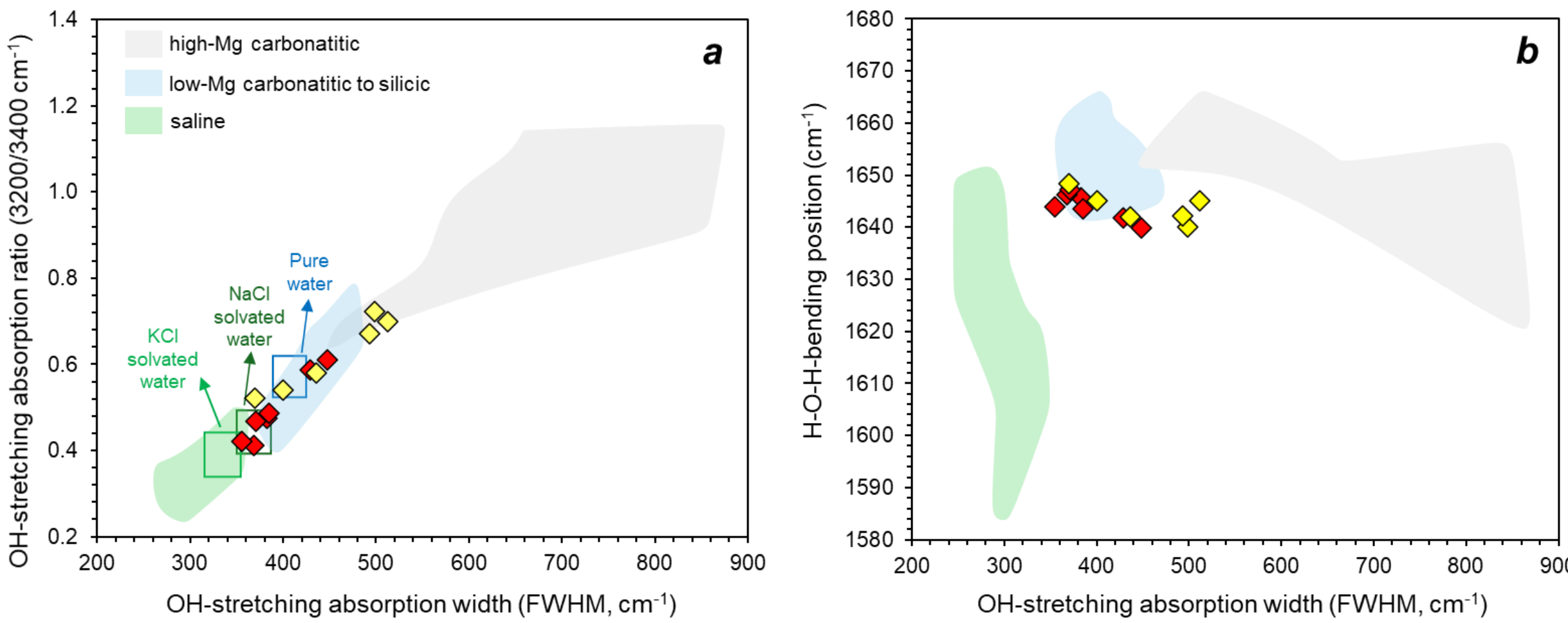

Supplementary Figure 2. a) OH-stretching absorption ratio (3200/3400 cm^{-1}) plotted as a function of the FWHM (cm^{-1}) of the OH-stretching absorption peak showing fields corresponding to high-Mg carbonatitic, low-Mg carbonatitic – silicic and saline HDFs in addition to fields for KCl and NaCl solvated water and pure water. **b**) The position of the H-O-H (H_2O) bending peak (cm^{-1}) plotted as a function of the FWHM (cm^{-1}) of the OH-stretching absorption peak. Data points overlap mostly with the low-Mg carbonatitic – silicic field indicating this HDF type for the fluid-rich zone of diamond K-09. Red and yellow diamonds indicate data from spectra recorded from the darker zones b-1 and b-2 and the lighter zones b-3 and b-4 (see Supplementary Table 1 and Fig. 5). Plots are modified from Weiss et al.[1].

EPMA data from HDFs in the fluid-rich zone of diamond K-09

$\delta^{13}C$ data from gem-quality core zone and fluid-rich zone

Supplementary Table 2. $\delta^{13}C$ data from gem-quality core (zone a), fluid-rich rim (zone b) and gem-quality outer rim (zone c) of diamond K-09. $\delta^{13}C$ data for A1, A2 and A3 asteriated diamonds (Marange, Zimbabwe) are also reported.

K-09			
fragment	zone	$\delta^{13}C$ (± 0.19)	Weight (mg)
gem(core)	a	-6.61	0.038
gem(core)	a	-5.72	0.039
gem(core)	a	-6.17	0.025
fluid-rich	b	-7.14	0.048
fluid-rich	b	-7.76	0.034
gem(rim)	c	-7.84	0.048
gem(rim)	c	-7.80	0.040

Asteriated diamonds			
		$\delta^{13}C$ (± 0.19)	Weight (mg)
A1		-9.25	0.055
A2		-9.04	0.033
A3		-8.86	0.032

References:

1. Weiss, Y., Kiflawi, I., & Navon, O. IR spectroscopy: quantitative determination of the mineralogy and bulk composition of fluid microinclusions in diamonds. *Chem. Geol.* **275**, 26–34 (2010).
2. Goss, J. P., Briddon, P. R., Hill, V., Jones, R., & Rayson, M. J. Identification of the structure of the 3107 cm−1 H-related defect in diamond. *J. Phys. Condens. Matter* 26, 145801 (2014).
3. Day, M. C., Jollands, M. C., Novella, D., Nestola, F., Dovesi, R. & Pamato, M. G. Hydrogen-related defects in diamond: A comparison between observed and calculated FTIR spectra. *Diam. Relat. Mater.* 143, 110866 (2024).
4. Howell, D., Weiss, Y., Smit, K. V., Loudin, L., & Nestola, F. DiaMap: New applications for processing IR spectra of fluid-rich diamonds and mapping diamonds containing isolated nitrogen (Type Ib) and boron (Type IIb). *In International Kimberlite Conference: Extended Abstracts*, Vol. 11 (2017).
5. Thompson, W. K. Infra-red spectroscopic studies of aqueous systems. Part 1.—Molar extinction coefficients of water, deuterium oxide, deuterium hydrogen oxide, aqueous sodium chloride and carbon disulphide. *Trans. Faraday Soc.* **61**, 2635–2640 (1965).
6. Venyaminov, S. Y., & Prendergast, F. G. Water (H_2O and D_2O) molar absorptivity in the 1000–4000 cm^{-1} range and quantitative infrared spectroscopy of aqueous solutions. *Anal. Biochem.* **248**, 234–245 (1997).
7. Day, M. C. et al. An updated and expanded Type classification scheme for diamonds based on FTIR spectroscopic data. *Am. Mineral.* (10.2138/am-2026-10296) (2026).
