# Supplementary material for: Nanometric mineral inclusions from a fluid-rich diamond: identification, structure, and implications for deep Earth
Source: Nat Commun. 2026 Jun 23;17:7915. doi: 10.1038/s41467-026-74619-3 (PMC13443600; doi:10.1038/s41467-026-74619-3)
Supplement: Supplementary file 2 [file 41467_2026_74619_MOESM2_ESM.pdf]

Description of Additional Supplementary Files

File Name: Supplementary Data 1
Description: Electron microprobe analyses of the silicic to low-Mg carbonatitic HDFs in fluid-rich diamond K-09
