# Supplementary material for: Nanometric mineral inclusions from a fluid-rich diamond: identification, structure, and implications for deep Earth
Source: Nat Commun. 2026 Jun 23;17:7915. doi: 10.1038/s41467-026-74619-3 (PMC13443600; doi:10.1038/s41467-026-74619-3)
Supplement: Supplementary file 3 [file 41467_2026_74619_MOESM4_ESM.pdf]

nature portfolio

Peer Review File

Nanometric mineral inclusions from a fluid-rich diamond: identification, structure, and implications for the deep Earth

Corresponding Author: Professor Fabrizio Nestola

This file contains all reviewer reports in order by version, followed by all author rebuttals in order by version.

Version 0:

Reviewer comments:

Reviewer #2

(Remarks to the Author)
Dear authors and editor,

Thank you for giving me the opportunity to read this interesting manuscript.

While I do not consider myself a specialist in diamond geology or micro-electron diffraction, I approach this manuscript as a researcher working at the nanoscale using complementary analytical techniques. My comments should therefore be understood as those of a colleague with relevant experience in nanometric materials characterization, rather than as an expert assessment of diamond petrology or MED methodology.

This manuscript reports the first application of MED to nanometric mineral inclusions extracted from a fluid-rich diamond, leading to the structural identification and anisotropic refinement of åkermanite, as well as the identification of several carbonate and fluoride phases. The authors combine MED with FTIR spectroscopy to infer the composition of high-density fluids and propose two scenarios for åkermanite crystallization related to the decompression of diamond-hosted fluids.

The study is technically innovative, addresses an analytical limitation in the study of fibrous diamonds, and provides new mineralogical constraints on diamond-forming media. The interpretations are plausible.

The analytical novelty is high, and the work clearly advances beyond prior TEM-SAED-based identification attempts. The authors have done an excellent job, and I welcome the use of new techniques to push the boundaries of what can be achieved in our field.

As an experimentalist, I naturally had some concerns about the combustion approach used for sample preparation for MED analysis (e.g., phase decomposition or oxidation of material). The authors explicitly address the possibility of artefact mineral formation during combustion and argue convincingly that åkermanite is not a combustion product. The thermal constraints cited are reasonable. If I may suggest one point for improvement, I would encourage the authors to further strengthen this argument, although the current discussion is already satisfactory.

In conclusion, the manuscript is technically sound, presents high-quality data, and has clear implications for mantle fluid processes. It should be of broad interest to geochemists, mineralogists, and Earth scientists. I recommend acceptance and invite the authors to consider expanding their discussion of potential artefacts.

Danilo Di Genova

Reviewer #3

(Remarks to the Author)
Noteworthy results?:
There are three novel results claimed by the authors here, the first anisotropic structure refinement of a nanometer sized inclusion from a fluid-rich diamond using MED; the first report of akermanite in diamond; and the identification of Ca-rich strontianite. While the manuscript and the work reported seems technically excellent, my concerns are that this is a single-

sample study with ambiguous geologic interpretation, and it appears to be a technical incremental refinement rather than a transformative breakthrough. The work will, I think, be primarily of interest to diamond inclusion specialists and in the end we are left with the impression that we cannot distinguish between two competing formation scenarios.

Regarding the akermanite report, it is novel, but one is left wondering what the significance is since it does not fundamentally change our understanding unless it provides unique constraints. Many minerals have been found in diamonds, and one is left with the impression that one more has been added to the list. As far as the Ca-rich strontianite-II us concerned, this is interesting, but is identified in only three grains from the same same so it is unclear whether this is geologically significant or simply a local curiosity.

Significance to the field?
The authors demonstrate that MED can work on nanometric diamond inclusions, providing a template for future studies, but the single sample study fails to establish systematic payments and the geologic interpretation is too ambiguous to guide future work. There seem to be no new constraints on mantle composition or processes, the formation scenarios for akermanite are equivocal, and the P-T constraints are neither novel nor particularly tight. We are not left with any significant new insights into diamond formation processes or the timing or mechanisms of cratonic evolution, for example. Neither does the paper touch significantly on deep Earth volatile cycles. This constrasts with, for example, Weiss et al (2021) Nature Comms where multiple samples gave and He isotope measurements had significant impact in terms of the development of a temporal framework for cratonic evolution.

Claims supported?
The authors present two scenarios for akermanite formation but cannot distinguish between them. If it crystallised during kimberlite eruption the entire thermochronometric argument (lines 375-403) would seem to be invalid and the "minimum temperature of 1000oC at 140km depth" is meaningless. If akermanite crystallised during pre-eruption exhumation the thermochrometry works but the timing of metasomatism remains unknown and the P-T constraints are relatively weak. The single sample limitation is a further challenge. We are left uncertain whether akermanite is common or unique, if Sr-Ba enrichment is regional or sample specific, and whether the formation scenarios apply broadly or just locally.

Data?
The structure refinements are solid, but with an R-factor of 13.5% I would class them as acceptable rather than excellent. For a reliable and stable anisotropic crystal structure refinement, the recommended ratio of observed independent reflections to refined parameters is typically greater than 8:1 for non-centrosymmetric structures and greater than 10:1 for centrosymmetric structures, but here we are told it is >5, so one assumes that it is close to 5, marginal for anisotropic refinement. This is born out by the problems in refinement of O2, with negative U33. Could this be a result of partial occupancy or some other form of disorder or systematic errors in the dynamical scattering model? Without this, the refined composition, in particular the placement of Fe^{2+} at the T2 position, becomes a question. The composition reported, $Si_{1.86}Fe^{2+}_{0.04}Al_{0.03}$, is assigned to match bond lengths yet the authors state that there is no evidence in the literature for Fe^{2+} at the T2 site. As far as bond lengths are concerned, all are reported in the narrative without any estimated standard deviation. Similarly no errors in the site occupancy values or e.p.s. values are given in the text.

Methodology?
The technical aspects of the paper appear sound, but without application to more than this one sample and effectively one data point, the transferability or wider impact of both the methodology and the results will remain uncertain.

Conclusion:
This is good, careful work that demonstrates technical capability, but it doesn't have the scope, clarity, or impact for Nature Communications. While a technical advance, this paper does not provide significant new insight into deep Earth processes, or diamond formation, by virtue of the ambiguity in the interpretations and the limited sample scope. I feel that the impact is commensurate with publication in more specialist literature than that expected by the broad readership of Nature Communications,

Version 1:

Reviewer comments:

Reviewer #3

(Remarks to the Author)
I've reviewed the authors' rebuttal along with the original reviewer comments. The authors have added significant new analyses that directly address the main concern about limited scope and ambiguous geological interpretation. In particular the new EMPA data showing elevated BaO, Cl, and K_2O in HDFs, support the saline fluid contribution from subducted oceanic crust hypothesis and the C isotopic data across all three zones support separate growth events and altered oceanic crust involvement. These data make the tectonic implications clearer. Nice. The case for pre-eruptive exhumation is also made clear. The crystallographic aspects represent foundational study and the authors make a strong case for this. They establish a method for P-T-fO2 reconstruction that others will likely follow.

In view of this I feel that the manuscript is now acceptable for publication in Nature Communications. It is now more clear that it represents a solid contribution that advances both analytical capabilities and understanding of cratonic diamond formation.

One final suggestion for further improvement is that the authors strengthen their case by adding a brief statement in the revised Discussion explicitly comparing their K-09 findings to other Kaapvaal fibrous diamonds with similar HDF compositions (they mention this in the rebuttal but should ensure it's prominent in the manuscript itself).

Open Access This Peer Review File is licensed under a Creative Commons Attribution 4.0 International License, which permits use, sharing, adaptation, distribution and reproduction in any medium or format, as long as you give appropriate credit to the original author(s) and the source, provide a link to the Creative Commons license, and indicate if changes were made.
In cases where reviewers are anonymous, credit should be given to 'Anonymous Referee' and the source.
The images or other third party material in this Peer Review File are included in the article's Creative Commons license, unless indicated otherwise in a credit line to the material. If material is not included in the article's Creative Commons license and your intended use is not permitted by statutory regulation or exceeds the permitted use, you will need to obtain permission directly from the copyright holder.
To view a copy of this license, visit https://creativecommons.org/licenses/by/4.0/

May 8th, 2026

Below we have provided detailed responses (given in blue font) to both reviewers. We have done out best to describe what modifications have been made to the manuscript and how the additional EMPA and EA-IRMS $\delta^{13}C$ data was used to strengthen our conclusions.
All revisions and additions are indicated in the "Text_R1_track_changes.docx" version of the revised manuscript.

==

Reviewer #1 (Remarks to the Author):

Dear authors and editor,

Thank you for giving me the opportunity to read this interesting manuscript.

While I do not consider myself a specialist in diamond geology or micro-electron diffraction, I approach this manuscript as a researcher working at the nanoscale using complementary analytical techniques. My comments should therefore be understood as those of a colleague with relevant experience in nanometric materials characterization, rather than as an expert assessment of diamond petrology or MED methodology.

This manuscript reports the first application of MED to nanometric mineral inclusions extracted from a fluid-rich diamond, leading to the structural identification and anisotropic refinement of åkermanite, as well as the identification of several carbonate and fluoride phases. The authors combine MED with FTIR spectroscopy to infer the composition of high-density fluids and propose two scenarios for åkermanite crystallization related to the decompression of diamond-hosted fluids.

The study is technically innovative, addresses an analytical limitation in the study of fibrous diamonds, and provides new mineralogical constraints on diamond-forming media. The interpretations are plausible.

The analytical novelty is high, and the work clearly advances beyond prior TEM-SAED-based identification attempts. The authors have done an excellent job, and I welcome the use of new techniques to push the boundaries of what can be achieved in our field.

As an experimentalist, I naturally had some concerns about the combustion approach used for sample preparation for MED analysis (e.g., phase decomposition or oxidation of material). The authors explicitly address the possibility of artefact mineral formation during combustion and argue convincingly that åkermanite is not a combustion product. The thermal constraints cited are reasonable. If I may suggest one point for improvement, I would encourage the authors to further strengthen this argument, although the current discussion is already satisfactory.

In conclusion, the manuscript is technically sound, presents high-quality data, and has clear implications for mantle fluid processes. It should be of broad interest to geochemists, mineralogists, and Earth scientists. I recommend acceptance and invite the authors to consider expanding their discussion of potential artefacts.

Danilo Di Genova
===
The authors greatly appreciate the review and constructive comments provided by Dr. Di Genova.

We are happy to hear that Rev#1 feels that we have provided sufficient information to prove that åkermanite is not a product of the combustion procedure. Although Rev#1 deems this argument "satisfactory", we offer the following synopsis of the related evidence provided in the manuscript:

[1] Melilite-group minerals (like åkermanite) with a composition similar to the åkermanite studied here have been previously synthesized at temperatures of 1000-1350 °C, at least 100 °C higher than the maximum temperatures attained during the combustion procedure (Akasaka et al. 2005; Aitasalo et al. 2007; Ardit et al. 2012).

[2] The temperature-composition phase diagram of Deer et al. (2012) shows formation of åkermanite, with a composition similar to that reported here (e.g., åkermanite 78% – gehlenite 22%), just below the solidus of the åkermanite–gehlenite binary join at temperatures lower than 1390 °C.

[3] Tavangarian et al. (2021) show that åkermanite may form at slightly lower temperatures (~900 °C) but only when multi-hour, high-energy ball-milling and long dwell times are employed during the synthesis procedure. As peak combustion temperatures of 900 °C were attained for only ~1 minute, it is clear åkermanite in this work could not have formed at this temperature.

[4] Sr, Ba- and alkali-bearing silicate and carbonate daughter phases have been previously identified (**via *in-situ* analyses**) as daughter-phases in HDFs in silico-carbonatitic fluid-rich diamonds (Weiss et al. 2022).

Reviewer #2 (Remarks to the Author):

Noteworthy results?:
There are three novel results claimed by the authors here, the first anisotropic structure refinement of a nanometer sized inclusion from a fluid-rich diamond using MED; the first report of akermanite in diamond; and the identification of Ca-rich strontianite. While the manuscript and the work reported seems technically excellent, my concerns are that this is a single-sample study with ambiguous geologic interpretation, and it appears to be a technical incremental refinement rather than a transformative breakthrough. The work will, I think, be primarily of interest to diamond inclusion specialists and in the end we are left with the impression that we cannot distinguish between two competing formation scenarios.
==

We thank Rev#2 for his/her insightful and constructive comments, it is clear that Rev#2 conducted an extremely detailed and careful review of our paper and we believe that the corresponding revisions have significantly improved the paper.

Detailed responses are provided below for all comments/concerns raised by Rev#2. All changes have been explicitly marked in the attached version of the manuscript "Text_R1_track_changes.docx".

Regarding the akermanite report, it is novel, but one is left wondering what the significance is since it does not fundamentally change our understanding unless it provides unique constraints. Many minerals have been found in diamonds, and one is left with the impression that one more has been added to the list. As far as the Ca-rich strontianite-II us concerned, this is interesting, but is identified in only three grains from the same same so it is unclear whether this is geologically significant or simply a local curiosity.
==

While it is true that many different (often larger) minerals (as defined by both structural - crystallographic and chemical analyses) have been identified in gem-quality lithospheric diamonds (e.g., Stachel et al. 2022), far fewer minerals have been *rigorously identified* in fibrous or fluid rich diamonds. As described in the introduction and discussion, such "identification" in fluid-rich diamonds is often based on chemical (e.g., EDS) or spectroscopic data from multiphasic inclusions allowing only general (or ambiguous) identification, e.g., as "carbonate" where differentiation amongst the hundreds of known carbonates (e.g., norsethite, strontianite and nyerereite as identified here) requires crystallographic data. Although general (bulk) chemical data from inclusions in fibrous diamonds may broadly reflect the composition or sources of parental fluids/melts, further work on reconstructing the P/T/fO_2 conditions of fluid rich diamond formation will absolutely require that inclusion phases can be identified using crystallographic data in additional to chemical data (again, we remark it is impossible to identify the inclusion only by chemical data).

Regarding the local or regional significance of the inclusions studied here, Sr-, Ba- and CO_3-bearing HDFs are commonly observed in fibrous diamonds from a variety of localities (e.g., Weiss et al. 2014, 2022, 2023; Zedgenizov et al. 2011; Smith et al. 2011; Kopylova et al. 2010; Klein-BenDavid et al. 2009) and are likely a typical residual product of carbonatitic diamond-forming fluids rather than a local curiosity. Most importantly, the identification of similar carbonate inclusions (e.g., nyerereite) in gem-quality lithospheric and sublithospheric

diamonds (Howell et al. 2012; Kaminsky et al. 2009) highlights possible similarities in the parental carbonatitic fluids/melts of both gem-quality and fibrous diamonds. **In the revised discussion, compositional similarities between the HDFs (and the minerals they contain) in sample K-09 and those in other carbonatitic fibrous diamonds have been highlighted (based on the new EMPA data, see below).**

Significance to the field?

The authors demonstrate that MED can work on nanometric diamond inclusions, providing a template for future studies, but the single sample study fails to establish systematic payments and the geologic interpretation is too ambiguous to guide future work. There seem to be no new constraints on mantle composition or processes, the formation scenarios for akermanite are equivocal, and the P-T constraints are neither novel nor particularly tight. We are not left with any significant new insights into diamond formation processes or the timing or mechanisms of cratonic evolution, for example. Neither does the paper touch significantly on deep Earth volatile cycles. This constrasts with, for example, Weiss et al (2021) Nature Comms where multiple samples gave and He isotope measurements had significant impact in terms of the development of a temporal framework for cratonic evolution.

Unifying models of deep Earth volatile cycling and diamond formation rely on aggregated evidence, but are only as accurate as the data on which they are based. Unambiguous, crystallographically constrained data at the nanoscale provides a means for future studies to produce unique, higher-confidence data and thus more representative models based on such data. In other words, we consider this work an attempt to better constrain, rather than generalize, data from fibrous diamonds, an exercise that we consider extremely important, especially in a field where the nanometric nature of the minerals being studied has resulted in significant ambiguity.

The crystallographic study of nanometric inclusions in diamonds (particularly daughter-phases as described here) will likely follow the same trajectory as the historical study of large inclusions in gem diamonds. Such work, initially conducted in the 1950-1960s, was largely descriptive, yet was still considered high-impact (e.g., Meyer, 1968, published in *Science*), and it was not until more samples (inclusions) from different localities were studied, that more universal insights into diamond formation and craton evolution could be made. Although the overall suite of primary inclusions captured by fibrous diamonds is likely similar to those observed in gem-quality diamonds (e.g., olivine, pyroxene, garnet, etc.), preliminary studies have revealed a huge variety of daughter-phases (carbonates, phosphates, silicates, oxides, sulphides, and residual LDFs). Until much more, higher-quality data from such daughter phases is collected (e.g., via MED), reconstructing the parental fluids from which they crystallized, and associated P/T/fO_2 conditions, will remain an extremely challenging task. We believe that the MED methods developed in this work will serve as the basis for a variety of impactful crystallographic work on mineral inclusions in diamonds in the future.

However, in an effort to draw broader and more novel conclusions about diamond formation and related metasomatic processes associated with the evolution of the Kaapvaal craton, the following additional data was collected from sample K-09;

[1] Major element contents of HDF inclusions from the fluid-rich (fibrous) zone.

EMPA analysis was conducted on the fluid-rich zone of the same crystal fragment (xtl-08, see Fig. 2) analysed via FTIR spectroscopy. Major element data revealed a low-Mg carbonatitic to silicic composition, in agreement with the data from FTIR spectroscopy and the suite of carbonates identified via MED. This new EMPA data has been integrated into the paper (e.g., see new Figures 3 and 4). Very interestingly, BaO, Cl and K_2O contents are relatively higher than expected for low-Mg carbonatitic – silicic HDFs implying a fluid sourced from seawater-altered oceanic crust and/or sea-floor sediments in subducting slabs. In-turn, this allowed us to add more detail to the discussion to the paper in which subduction (possibly associated with the Proterozoic Namaqua-Natal Orogeny) resulted in the percolation of saline fluids through a carbonated eclogite and the subsequent production of a parental silicio-carbonatitic fluid enriched in BaO, Cl, SrO and K_2O. This observation provides important additional constraints on a mixed-source for carbonatitic metasomatic fluids affecting the Kaapvaal cratonic mantle that have been previously linked to eclogitic diamond formation (e.g., Richardson et al. 2004).

[2] ^{13}C isotopic composition of both the gem-quality core zone and fluid-rich rim zone.

Bulk ^{13}C isotopic analyses have been conducted for each of the three zones (*a*, *b* and *c*) that have been identified via FTIR. ^{13}C isotopic analyses are a fundamental tool to understand the origin of the diamonds and the nature of the diamond-forming media itself, and can help to differentiate distinct diamond growth events and the subsequent evolution of the parental fluid/melt during crystallization. The new data has confirmed differences between the gem-quality core and the fluid-rich rim: the core has a ^{13}C value that overlaps with the lower end of the normal mantle ^{13}C array, while the fluid-rich rim is characterized by more negative values, which suggest two different growth events. Moreover, the more negative ^{13}C isotopic composition of the fluid-rich rim represents a crucial constraint on the involvement of recycled crustal material (altered oceanic crust) in the lithospheric mantle beneath the Kaapvaal Craton. This data further supports that subduction was related to the metasomatic event that resulted in formation of the fluid rich zone of diamond K-09.

Claims supported?
The authors present two scenarios for akermanite formation but cannot distinguish between them. If it crystallised during kimberlite eruption the entire thermochronometric argument (lines 375-403) would seem to be invalid and the "minimum temperature of 1000oC at 140km depth" is meaningless. If akermanite crystallised during pre-eruption exhumation the thermochrometry works but the timing of metasomatism remains unknown and the P-T constraints are relatively weak. The single sample limitation is a further challenge. We are left uncertain whether akermanite is common or unique, if Sr-Ba enrichment is regional or sample specific, and whether the formation scenarios apply broadly or just locally.

Two possible scenarios are described in the discussion for the formation of åkermanite from HDFs upon depressurization due to [1] kimberlite eruption and [2] exhumation of diamond K-09 prior to eruption. For the sake of transparency, both scenarios are described, however, it is highly likely that åkermanite formed during exhumation rather than kimberlite eruption, relevant sections of the discussion have been revised to better highlight this point.

In a recent paper (Wincott et al. 2026), based on FTIR-thermochronometric analysis of diamonds (the same methods used to defend scenario [2]), a mantle cooling rate of 42-62 °C/Gyr is proposed, making scenario [2] very likely. As stated in the revised discussion, a difference in the $Temp_{res}$ between the core and fluid-rich rim of 75-175 °C is required to explain the observed N-aggregation states of the core and rim. It is important to note, this range was calculated using the maximum $Temp_{res}$ of the fluid-rich rim and thus represents the minimum $Temp_{res}$ difference between the core and rim. The true $Temp_{res}$ difference is likely significantly larger. Even if this minimum $Temp_{res}$ difference is used, it can only be explained assuming normal mantle cooling (scenario [1]) if the fluid rich rim formed earlier than ~1 Ga. We propose in the discussion this is unlikely based on similarities with the ages of other Proterozoic eclogitic diamonds from the Kaapvaal and the timing of subduction associated with the Namaqua-Natal Orogeny, now invoked to explain the apparent saline contribution based on EMPA data.

We believe the new data strongly support scenario [2], as a complex and multi-stage history must be already invoked to explain the geochemical and isotopic features of diamond K-09.

In the revised discussion, evidence supporting åkermanite formation due to exhumation prior to kimberlite eruption is described in more detail. This is also strongly supported by the work of Nimis et al. (2025) who describe similar processes for gem-quality diamonds from the same locality (Kimberley region). Using elastic (thermo)barometric data from touching and non-touching mineral inclusions, these authors show that such diamonds were transported tens of kilometres upwards during multi-stage mantle uplift associated with Archean tectonism. As the core of diamond K-09 is likely Archean in age, the model from Nimis et al. (2025) supports exhumation of diamond K-09 prior to kimberlite eruption as suggested by the FTIR thermochronometric data.

Consequently, we feel that the minimum temperature of diamond formation based on the observation of åkermanite (1000 °C at 4.6 GPa and 140km depth) is reasonable.

The potential abundance of åkermanite and Sr- and Ba-enrichment in fibrous diamonds is addressed in the response above.

Data?
The structure refinements are solid, but with an R-factor of 13.5% I would class them as acceptable rather than excellent. For a reliable and stable anisotropic crystal structure refinement, the recommended ratio of observed independent reflections to refined parameters is typically greater than 8:1 for non-centrosymmetric structures and greater than 10:1 for centrosymmetric structures, but here we are told it is >5, so one assumes that it is close to 5, marginal for anisotropic refinement. This is born out by the problems in refinement of O2, with negative U33. Could this be a result of partial occupancy or some other form of disorder or systematic errors in the dynamical scattering model? Without this, the refined composition, in particular the placement of Fe2+ at the T2 position, becomes a question. The composition reported, Si1.86Fe2+0.04Al0.03, is assigned to match bond lengths yet the authors state that there is no evidence in the literature for Fe2+ at the T2 site. As far as bond lengths are concerned, all are reported in the narrative without any estimated standard

deviation. Similarly no errors in the site occupancy values or e.p.s. values are given in the text.

The reviewer claims that an R-factor of 13.5% is acceptable rather than excellent. We agree, but only in the case where one is dealing with conventional X-ray diffraction (kinematic) analysis of large crystals (tens to hundreds of microns). Instead, here we present data on electron diffraction (highly dynamic interaction) on a crystal which is 1000 times smaller!

The referee can surely refer to the recent paper in IUCrJ by Gemmi et al. (2026) in which seven laboratories performed round robin testing using different transmission electron microscopes on selected materials, including a mineral (epidote) with a composition not far from the one we dealt with in this manuscript. Gemmi et al. (2026) concluded that dynamical refinement reduces significantly the refinement *R* factors and improves the accuracy of the structure models providing fine structural details (like the ones discussed in this manuscript). In the case of epidote, the best result they obtained is R(all) of 9.68%, whereas the average among the seven labs was 12.11%. The lowest R was obtained combining precession (PEDT) plus continuous rotation (cRED), whereas we used only cRED. The average standard deviation for epidote in the round robin was 0.0106 Å at best, and 0.026 Å on average. However, we cannot perform the same approach because we cannot compare the model obtained by single crystal X-ray diffraction by using the *COMPSTRU* utility of the Bilbao Crystallographic Server (we do not have a suitably large crystal to allow for a comparison). Yet, the data reported in Table 2 shows that we have errors in individual bond distances < 0.01 Å (which is absolutely an extremely good result considering the nanometric size of the crystal).

The reduced ratio of observed independent reflections to refined parameters is due to the angular range in which we collected data, which was reduced to 80° due to the unfortunate position of the interesting nanometric grain (very close to the copper grid). Round robin results by Gemmi et al. (2026) are obtained with angular ranges of 110-140°. Only in one case was this reduced to 60° and, in that case, the team was unable to perform dynamical refinement and obtained R(all) of 24.55%. Access to the reciprocal space is intrinsically limited by the support when performing electron diffraction, which is not the case when dealing with standard X-ray techniques.

Considering the above information, we believe that we reached a very good result with the structure refinement described here.

We must humbly disagree with referee when he states that we have not provided standard deviations for site occupancies (see Table 2 and CIF file). The text reports many values with corresponding standard deviations (including angles). We admit that in three cases (reporting m.a.n; which are calculated from refined site occupancies, for which we reported standard deviations are reported anyway), standard deviations were not provided and therefore we have now added them. Standard deviations can be reported for single bond distances but are not applicable to the average bond distances, this is never done for standard X-ray diffraction publications. Calculated bond distances and m.a.n. are provided without any standard deviation.

Ca m.a.n. 23.6(6) e.p.s.
Mg (T1) m.a.n. 14.0(7) e.p.s.
Si (T2) m.a.n. 14.4(4) e.p.s.

About the Fe^{2+} at the T site, we would like to remind to the reviewer that this is just a hypothesis. Nevertheless, the amount of Fe^{2+} we are considering here is so small that it does not make any difference in the overall structure refinement and we cannot provide more rigorous evidence of its occupancy at the T site based on crystal chemical grounds.

Methodology?
The technical aspects of the paper appear sound, but without application to more than this one sample and effectively one data point, the transferability or wider impact of both the methodology and the results will remain uncertain.

Following our response to Rev# 2's concerns (see "Significance to the field?" above), we believe that the potential impact of this paper, in particular the transferability of the methods (i.e., MED) utilized, is clear.

We have shown that by using electron diffraction, information can be extracted from diamonds at the nanometric scale providing access to a completely new repository of information. Admittedly, the total extent to which such crystallographic data can be used to improve our understanding of diamond formation will remain unknown until more related studies are completed. However, the authors do not believe that uncertainty regarding the array of potential geological applications makes this works less novel and/or impactful.

Conclusion:
This is good, careful work that demonstrates technical capability, but it doesn't have the scope, clarity, or impact for Nature Communications. While a technical advance, this paper does not provide significant new insight into deep Earth processes, or diamond formation, by virtue of the ambiguity in the interpretations and the limited sample scope. I feel that the impact is commensurate with publication in more specialist literature than that expected by the broad readership of Nature Communications,

In an effort to address the major concerns of Rev#2 regarding the implications about deep Earth processes and diamond formation, additional EMPA and carbon isotopic data has been included and major changes have been made to the manuscript accordingly (see previous comments).

Based on the major revisions as described above, we think the revised manuscript serves as a guide for how coupling crystallographic, spectroscopic, major element geochemical and isotopic data can provide novel insights into fluid-rich diamond formation and associated metasomatic processes.

Regarding specifically the application of MED to the study diamond formation, we believe that this manuscript provides a foundation from which [1] more productive methodologies

can be developed for studying small mineral inclusions in fibrous diamonds, [2] comparing data from large mineral or fluid inclusions in gem-quality diamonds to analogous data from nanometric inclusions in fibrous diamonds, and [3] linking data from inclusions, in both fibrous and gem-quality diamonds, to common parental deep earth fluids/melts.

Sincerely,

Fabrizio Nestola and co-authors

References:

Aitasalo, T., Hölsä, J., Kirm, M., Laamanen, T., Lastusaari, M., Niittykoski, J., Raud, J., & Valtonen, R. Persistent luminescence and synchrotron radiation study of the $Ca_2MgSi_2O_7$: Eu^{2+}, R^{3+} materials. *Radiat. Meas.* **42**, 644–647 (2007).

Akasaka, M., Nagashima, M., Makino, K., & Ohashi, H. Distribution of Fe^{3+} in a synthetic $(Ca,Na)_2(Mg,Fe^{3+})Si_2O_7$ melilite. *J. Mineral. Petrol. Sci*. **100**, 229–236 (2005).

Ardit, M., Dondi, M., Merlini, M., & Cruciani, G. Melilite-type and melilite-related compounds: structural variations along the join $Sr_{2-x}Ba_xMgSi_2O_7$ ($0\leq x\leq 2$) and high-pressure behavior of the two end-members. *Phys. Chem. Miner.* **39**, 199–211 (2012).

Deer, W. A., Howie, R. A., & Zussman, J. An introduction to the rock-forming minerals. *The mineralogical society, London*, 498 pp. (2013).

Gemmi, M., Palatinus, L., Boullay, P., Abrahams, J. P., Ben Meriem, A., Cordero-Oyonarte, E., Emerson Agbemeh, V., Chintakindi, H., Faye Diouf, M. D., Filipcik, P., Gemmrich Hernandaz, L., van Genderen, E., Hadermann, J., Hajizadeh, A., Jeriga, B., Kolb, U., Matinyan, S., Passuti, S., Santucci, M., Suresh, A., Parez, O., Tai, C.-W., Vypritskaia, A., Wang, L., Xu, & H., Zou, X. Round robin on structure analysis from 3D electron diffraction data. *IUCrJ*, ***13***, 198-210 (2026).

Howell, D., Wood, I. G., Nestola, F., Nimis, P., & Nasdala, L. Inclusions under remnant pressure in diamond: a multi-technique approach. *Eur. J. Mineral*. **24**, 563–573 (2012).

Kaminsky, F., Wirth, R., Matsyuk, S., Schreiber, A., & Thomas, R. Nyerereite and nahcolite inclusions in diamond: evidence for lower-mantle carbonatitic magmas. *Mineral. Mag.* **73**, 797–816 (2009).

Klein-BenDavid, O., Logvinova, A.M., Schrauder, M., Spetius, Z.V., Weiss, Y., Hauri, E.H., Kaminsky, F.V., Sobolev, N.V., & Navon, O. High-Mg carbonatitic microinclusions in some Yakutian diamonds—a new type of diamond-forming fluid. *Lithos* **112**, 648–659 (2009).

Kopylova, M., Navon, O., Dubrovinsky, L., & Khachatryan, G. Carbonatitic mineralogy of natural diamond-forming fluids. *Earth Planet. Sci. Lett.* **291**, 126–137 (2010).

Meyer, H. O. Chrome pyrope: an inclusion in natural diamond. *Science*, **160**, 1446-1447 (1968).

Nimis, P., Grütter, H.S. and Nestola, F. Multistage diamond formation, mantle uplift and changing geothermal regimes recorded by inclusions in Kimberley diamonds. *Mineralogy and Petrology* **119**, 413–432 (2025).

Richardson, S. H., Shirey, S. B., & Harris, J. W. Episodic diamond genesis at Jwaneng, Botswana, and implications for Kaapvaal craton evolution. *Lithos* **77**, 143-154 (2004).

Smith, E. M., Kopylova, M. G., Dubrovinsky, L., Navon, O., Ryder, J., & Tomlinson, E. L. Transmission X-ray diffraction as a new tool for diamond fluid inclusion studies. *Mineral. Mag*. **75**, 2657–2675 (2011).

Stachel, T., Aulbach, S. & Harris, J. W. Mineral Inclusions in Lithospheric Diamonds. *Rev. Mineral. Geochem*. **88**, 307–391 (2022).

Tavangarian, F., Zolko, C. A., & Davami, K. Synthesis, characterization and formation mechanisms of nanocrystalline akermanite powder. *J. Mater. Res. Technol.* **11**, 792–800 (2021).

Weiss, Y., Kiflawi, I., Davies, N., & Navon, O. High-density fluids and the growth of monocrystalline diamonds. Geochim Cosmochim Acta 141:145–159 Weiss Y, Kiflawi I, Davies N, Navon O (2014) High-density fluids and the growth of monocrystalline diamonds. *Geochim Cosmochim Acta* **141**, 145–159 (2014).

Weiss, Y., Czas, J., & Navon, O. Fluid inclusions in fibrous diamonds. *Rev. Mineral. Geochem.* **88**, 475–532 (2022).

Weiss, Y., Koornneef, J. M., & Davies, G. R. Sr-Nd-Pb isotopes of fluids in diamond record two-stage modification of the continental lithosphere. *Geochem Perspect Lett* ***27***, 20-25 (2023).

Wincott, M. M., Speich, L., Bulanova, G. P., Parkinson, I. J., Smith, C. B., & Kohn, S. C. Lithospheric mantle cooling recorded by zoned diamonds: forward modelling the nitrogen aggregation thermometer. *Earth Planet. Sci. Lett.* **680**, 119900 (2026).

Zedgenizov, D.A., Ragozin, A.L., Shatsky, V.S., Araújo, D., & Griffin, W.L. Fibrous diamonds from the placers of the northeastern Siberian Platform: Carbonate and silicate crystallization media. *Russ Geol Geophys* **52**, 1298–1309 (2011).
